# Diversity of estrogen biodegradation pathways and application in environmental bioremediation

**DOI:** 10.3389/fmicb.2025.1630636

**Published:** 2025-09-25

**Authors:** Jaleela S. Hashem, Wael Ismail, Yin-Ru Chiang, Adnan A. Bekhit

**Affiliations:** ^1^Allied Health Sciences Department, College of Health and Sport Sciences, University of Bahrain, Manama, Bahrain; ^2^Center of Environmental and Biological Studies, Arabian Gulf University, Manama, Bahrain; ^3^Biodiversity Research Center, Academia Sinica, Taipei, Taiwan; ^4^Department of Pharmaceutical Chemistry, Faculty of Pharmacy, Alexandria University, Alexandria, Egypt

**Keywords:** environment, wastewater, estradiol, pollution, endocrine-disrupting chemicals, 4,5-*seco* pathway

## Abstract

Steroid estrogens, including the naturally occurring hormones estrone (E1), estradiol (E2), and estriol (E3), as well as the synthetic estrogen ethinylestradiol (EE2), play essential physiological roles in the regulation of the reproductive systems and development of secondary sex characteristics in humans and animals. Environmental pollution with steroid estrogens is gaining rising concerns worldwide due to their endocrine-disrupting and carcinogenic properties, which can harm humans and aquatic organisms. Hence, efficient removal of these compounds, particularly from wastewater, is deemed key to prevent environmental pollution with estrogens. Although several physicochemical treatments contribute to estrogen elimination from wastewater treatment plants (WWTPs), biological treatment via microbial biodegradation remains the most efficient estrogen removal approach. Several estrogen-degrading/transforming bacteria were isolated mainly from activated sludge samples collected from WWTPs. Moreover, biochemical, and molecular aspects for estrogen degradation pathways were revealed recently for estrone and estradiol. On the contrary, less knowledge is currently available for E3 and EE2 biodegradation pathways. Despite high structural similarity among steroid estrogens, they can be degraded via a diversity of biodegradation and biotransformation pathways. Nonetheless, these pathways exhibit common as well as unique biochemical and molecular features. Moreover, steroid estrogens are interconvertible, which can affect their environmental concentrations, and hence, their persistence/biodegradability. In this review, we present and discuss the various steroid estrogen biodegradation and biotransformation pathways, with a focus on the biochemical aspects. Furthermore, we highlight some of the known abiotic estrogen reactions and the recent discoveries on microbial estrogenesis and envisage how they can affect estrogen susceptibility to microbial degradation.

## 1 Introduction

Environmental pollution by hazardous chemicals is one of the most critical global challenges. Some of these chemical pollutants can interfere with the natural functions of hormones in humans and animals, and hence are termed endocrine-disrupting chemicals (EDCs) (Zhang et al., 2015; Chiang et al., 2020; López-Velázquez et al., 2023; Lerdsuwanrut et al., 2025). The latter were defined by the World Health Organization (WHO) as “an exogenous substance or a mixture that alters function(s) of the endocrine system and consequently causes adverse health effects in an intact organism, or its progeny, or (sub) populations” (Bergman et al., 2012; Gonsioroski et al., 2020).

In the same context, scarcity of freshwater resources has become a global challenge that threatens sustainable development in many countries. This issue will escalate in the future due to the increasing global human population and urbanization and will be compounded by the negative impacts of climate change (Stikker, 1998; Stefanakis, 2022). Hence, reuse and recycling of wastewater from municipal, industrial, and agricultural sectors are being adopted as counter measures to increase the water resource base. However, wastewater streams require efficient treatment and remediation processes to remove hazardous pollutants, like EDCs, and ensure safe reuse (Stefanakis, 2018).

Estrogens are steroid hormones produced by humans and animals where they play key physiological roles in the regulation of the reproductive systems and development of secondary sex characteristics (Bilal et al., 2021). Although estrogens are necessary for humans and other vertebrates, chronic exposure to even trace amounts of estrogens (sub-nanomolar levels) can disrupt the endocrine system and sexual development (Kolodziej et al., 2003; Lee et al., 2006; Lambert et al., 2015; Regnault et al., 2018; Predieri et al., 2022). Hence, estrogens are considered EDCs and were classified by the WHO as group-1 carcinogens, and some catechol metabolites of estrogens were reported as carcinogens (Liehr et al., 1986; Fernandez et al., 2006; http://monographs.iarc.fr/ENG/Classification/latest_classif.php). These hazardous properties raised substantial concerns on environmental pollution with estrogens.

Steroid estrogens are represented by three naturally occurring hormones, including estrone (E1), estradiol (E2), and estriol (E3), as well as the synthetic estrogen ethinylestradiol (EE2) (Pratush et al., 2020a). Estrogens are found in aquatic environments and raise significant concerns on water and wastewater quality (Roudbari and Rezakazemi, 2018). They enter the environment via different routes, but mainly through discharge of urine and feces reaching wastewater treatment plants (WWTPs) (Adeel et al., 2017). Estrogens are commonly present in wastewater in trace amounts, ranging from a few ng/L to several μg/L, yet they are associated with adverse ecological impact and influence marine organisms in the impacted area, accumulating in the ecosystem and causing acute and chronic toxicity to organisms, as well as loss of habitat and biodiversity (Adeel et al., 2017; Chiang et al., 2020).

Estrogens accumulate in the tissues of fish and other marine organisms (Ros et al., 2016) and are associated with increased liver size, increased plasma glucose concentration, smaller gonad size, and less mature gametes. This may adversely disrupt the reproductive potential of marine organisms, reducing their numbers and thus causing decline in a sustainable food source for the exposed populations (Elliott et al., 2017). Likewise, the accumulation of estrogens seriously threatens humans and the food chain. Eventually, they build up in the human body and trigger health concerns, such as breast cancer in women and prostate cancer in men (Adeel et al., 2017). Consequently, effective elimination of estrogens and other EDCs from the environment has become an active research area.

Efficient removal of estrogens during wastewater treatment is essential to avoid environmental pollution. Physical removal methods such as reverse osmosis and adsorption on activated carbon do not eliminate the pollutants, albeit only transfer them from one phase to another. Moreover, chemical oxidation is not economically viable, and chemicals are reactive, and the process is associated with by-products with unknown effects (Koh et al., 2008; Bolong et al., 2009; Liu et al., 2009a). While several techniques, such as electrochemical oxidation, adsorption, photocatalysis, and membrane filtration, have been proposed for the removal of EDCs from the aquatic environment, yet they can be associated with high cost, operational complexity, and environmental impact (Ben et al., 2017).

On the contrary, microbial biodegradation is a green and economic process which plays a key role in estrogen removal from WWTPs (de Lorenzo, 2017). Several estrogen-degrading bacteria have been isolated mainly from activated sludge collected from WWTPs (Chiang et al., 2020). Despite the high structural similarity among the steroid estrogens, the reported biotransformation reactions and biodegradation pathways are very versatile. In this review, we first give a brief account on estrogens, their characteristics, how they reach and impact the environment and public health. Then, we explore and discuss the status quo of what is known about the microbial biodegradation of estrogens in terms of microbiology and diversity of the biodegradation pathways, highlighting knowledge gaps and proposing future research directions. We included early as well as recent studies reporting proposed biotransformation reactions and biodegradation pathways with more focus on bacteria. While we focus on the degradation and transformation metabolites and the flow of the pathways, we refer to involved key enzymes and genes whenever relevant. For more information on the genes and enzymes of estrogen degradation we refer the readers to several reviews (Olivera and Luengo, 2019; Chiang et al., 2020; Pratush et al., 2020a).

## 2 Estrogens: properties and physiological roles

**Figure 1:**
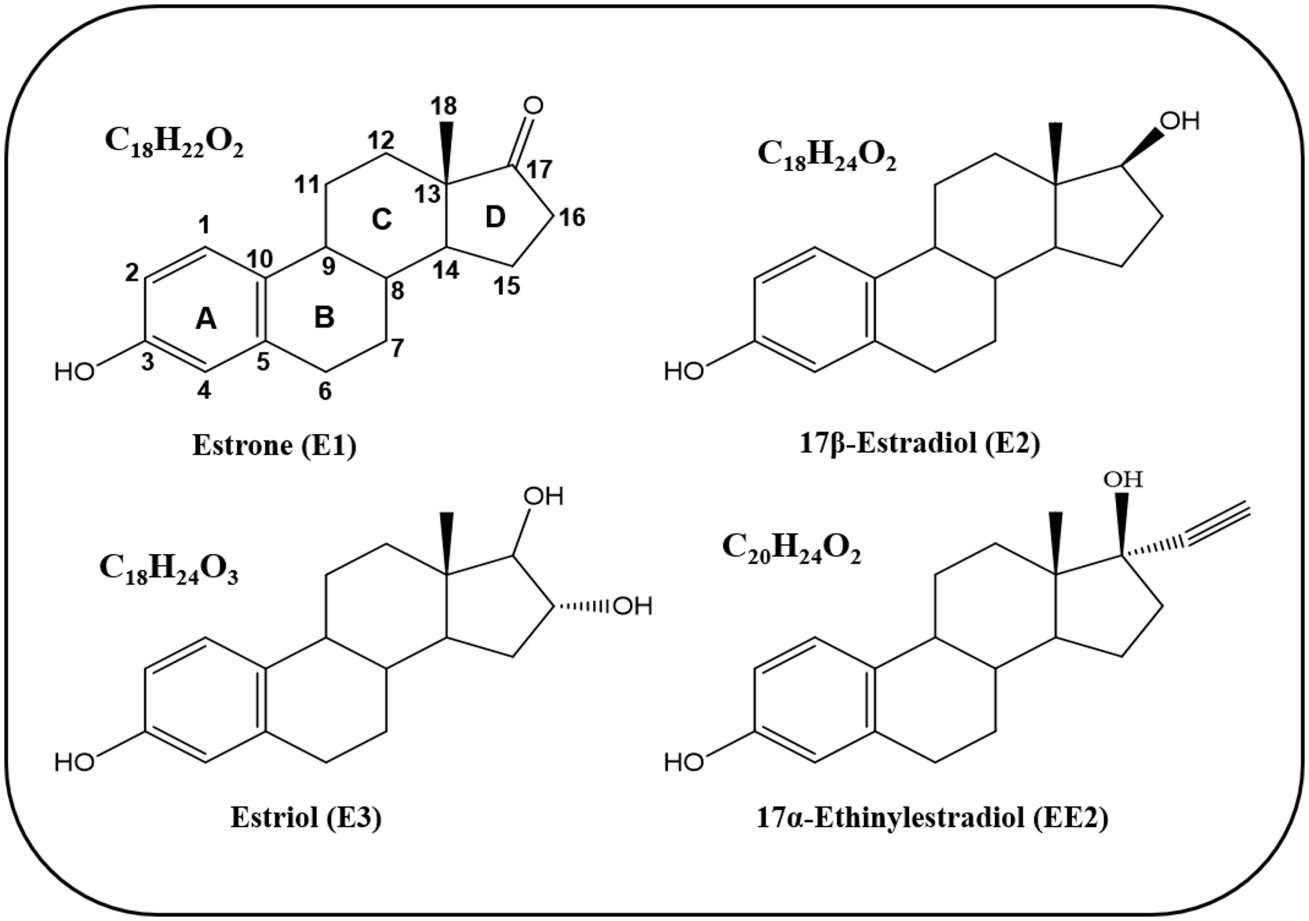
Chemical structure of natural and synthetic estrogens.

Estrogens are steroid hormones that function as signaling molecules, including natural estrogens such as estrone (E1), 17β-estradiol (E2), estriol (E3), as well as the synthetic estrogen 17α-ethinylestradiol (EE2) (Bilal et al., 2021). The core structure of estrogens comprises 18 carbon atoms and shares the same core molecular structure of sterane, tetracyclic framework, consisting of four fused rings (A, B, C, and D) (Figure 1) (Bilal et al., 2021). The A-ring is phenolic, B- and C-rings are cyclohexane, whereas the D-ring is a cyclopentane. The structural differences among estrogens lie in the configuration of the D-ring, specifically in carbons at positions 16 and 17.

The precursor of natural estrogens is dietary cholesterol (C_27_H_46_O), particularly the low-density lipoprotein (LDL) (Olivera and Luengo, 2019). In steroidogenesis, cholesterol is converted into sterane, a C_18_ steroid derivative, and the leading site of estrogen biosynthesis is the granulosa cells of the ovaries (Vazakidou et al., 2024). Yet, the translocation of cholesterol occurs in the inner membrane of the mitochondria and is regulated by a steroidogenic acute regulatory protein (StAR) (Lennartz et al., 2024). In the mitochondrial membrane, cholesterol is transformed to pregnenolone by the enzyme P450scc, which is further converted to androstenedione by the enzymes CYP17A1 and 3β-hydroxysteroid dehydrogenase (3β-HSD) (Cui et al., 2013). Androstenedione disperses to the granulosa cells across the basal lamina, where it produces E1 and E2 by the enzymes CYP19A1 (aromatase) and 17β-hydroxysteroid dehydrogenase (17β-HSD), respectively (Cui et al., 2013).

In addition to the well-known aromatase-dependent biosynthesis of C_18_ estrogens from C_19_ androgens, a very recent study showed that androgens can be transformed to estrogens in a Peptococcaceae bacterium isolated from the gut of the great, blue-spotted mudskipper (*Boleophthalmus pectinirostris*) (Wang et al., 2025). This bacterium, *Phosphitispora* sp. strain TUW77, has a novel testosterone fermentation pathway which can transform testosterone (androgen) into estrogens and androstanediol under anaerobic conditions. Upon growth on testosterone, the strain TUW77 strongly expresses a polycistronic gene cluster *abeABC* (anaerobic bacterial estrogenesis), encoding components of a classic cobalamin-dependent methyltransferase system, which enables the removal of the C19 methyl group of testosterone. The strain TUW77 then utilizes the removed methyl group as a carbon and electron source by oxidation to CO_2_ via the oxidative Wood-Ljungdahl pathway. Accordingly, Wang et al. (2025) suggested that the Wood-Ljungdahl pathway may have had a role in the emergence of estrogens in the early biosphere. These findings suggest that bacterial estrogenesis may have existed in nature prior to the O_2_-dependent production in vertebrates, which challenges the legacy that estrogen biosynthesis is restricted to aromatase-containing vertebrates. They also open new venues for research addressing the occurrence of this unusual phenotype within the microbial world, particularly in obligately anaerobic bacteria.

Natural estrogens are essential sex hormones that play important physiological roles in the development of the female reproductive system and secondary sexual characteristics (Fuentes and Silveyra, 2019) in addition to male external normal genitalia (Baskin et al., 2022). It is known that estrogens have roles in regulating immunity through their effect on the immune cells to prevent atherosclerosis and endothelial dysfunction by maintaining the endothelial cells and promoting angiogenesis (Trenti et al., 2018). The synthetic estrogen EE2 is commonly used in contraceptives. It is one of the most common medications for humans and animals and has raised various health issues and ecological concerns (Laurenson et al., 2014). It is a highly potent oral contraceptive and ranked among the top 15 US active ingredients in terms of its daily use (Kostich and Lazorchak, 2008).

## 3 Environmental pollution with estrogens

Humans and animals excrete steroid hormones in different amounts depending on age, state of health, diet, or pregnancy. Natural and synthetic estrogens are considered among the most significant pollutants in different environments, and the main sources of estrogens pollution are WWTPs, hospitals, pharmaceutical industry, agricultural manure, and effluents from livestock feedlots (Adeel et al., 2017; Chiang et al., 2020; López-Velázquez et al., 2021; Torres et al., 2021) (Table 1). Natural estrogens are free or conjugated with sulfate and/or glucuronide groups. Free estrogens are more hydrophobic with lower solubility than conjugated estrogens, even though they pose an estrogenic potential (Yu et al., 2019). After being metabolized in the body via conjugation with sulfate and hydroxylation reactions, the inactive forms of estrogens are excreted into the environment in urine and feces, which end up in WWTPs (Table 2).

**Table 1:** Steroid estrogen concentrations (ng/L) in influents and effluents of WWTPs and their removal percentage (Liu et al., 2009b, 2009c; Pratush et al., 2020a).

Estrogen	Country	Influent concentration	Effluent concentration	Removal (%)
Estrone	Japan	259–326	n.d.–17	93–100
	Italy	25–132	2.5–82	22–95
	Australia	29–670	n.d.–72	111–100
	USA	57.8–83.3	6.3–49.1	41–89
	Canada	n.d.–33	n.d.–147	n.a.
Estradiol	Japan	n.d.–57	4.6–14 b	100–92
	Italy	4–25	0.35–3.5	59–98
	Australia	35–125	n.d.–30	44–100
	USA	11.2–161.6	1.5–5.4	52–99
	Canada	2.4–26	0.2–14.7	18.5–98.8
Estriol	Japan	n.d.	n.d.–151	100–0
	Italy	24–188	0.43–18	77–99
	Australia	23–660	n.d.–275	18–100
	USA	79.7–259.2	2.2–3.9	95–98
	Canada	n.d.–22	n.d.–29	n.a.

n.d., not detected; n.a., data not available.

**Table 2:** Examples of concentrations (ng/L) of estrogen conjugates detected in WWTPs (Yu et al., 2019; Pratush et al., 2020a).

Estrogen	Conjugate	Influent concentration	Effluent concentration
Estrone	Estrone-3-sulfate	25	9
		42	13
		34.1	0.3
		18	11.8
		16.5	1.9
		76	27
		4.8	0.8
		4.4	0.7
	Estrone-3-glucuronide	4.3	0.7
		11	7.4
		0.4	n.d.–0.05
		0.6	n.d.–0.6
		3.6	n.d.
Estradiol	Estradiol-3-glucuronide	5.2	n.d.
		0.3	n.d.–0.07
		13.3	0.5
		2.0	0.4
		2.5	0.7
	Estradiol-17-glucuronide	18	91
		n.d.–0.25	n.d.-0.25
		10.5	n.d.–1.4
		51	21
		3	0.7
		3.6	0.9
	Estradiol-3-sulfate	3.3	n.d.
		110	52
		32	n.d.–0.07
		3.6	0.4
		2.7	1.8
		13	5.3
		5.5	0.7
		4.9	0.7
	Estradiol-3,17-disulfate	n.d.–0.28	n.d.–0.28
		28	3.3
Estriol	Estriol-3-glucuronide	22	72
		n.d.–0.28	n.d.–0.8
	Estriol-3-sulfate	14	2.2
	Estriol-17-glucuronide	30	n.d.

n.d., not detected.

In WWTPs, estrogens’ conjugates revert to free estrogens (active forms) through enzymatic reactions performed by microbes existing in the WWTPs (Palme et al., 1996; Liu et al., 2009c; Zhang et al., 2024). The presence of estrogens in aquatic ecosystems is hazardous to aquatic life due to their endocrine-disrupting activity, and the hazards may include synthesis and secretion of vitellogenin (a female specific protein) in male fish, development of intersex characteristics and/or failure in the development of normal secondary characteristics (Racz and Goel, 2010; Ying et al., 2002) which leads to changes in reproduction. In addition to their endocrine-disrupting activity, estrogens were listed by the WHO as “group 1″ carcinogens.

Moreover, some catechol metabolites of estrogens, such as 4-hydroxyestrone, were reported to exhibit carcinogenic activity (Liehr et al., 1986; Fernandez et al., 2006). Due to their biological activity at very low concentrations, EDCs can threaten the polluted environment with acute and chronic toxicity to organisms, loss of habitat and biodiversity, and a range of potential adverse effects on environmental and ecological health (Torres et al., 2021; Zheng et al., 2023).

In humans, studies have reported a close association between exposure to estrogens and disorders of the male and female reproductive systems. Estrogens can disrupt the hormone-dependent pathways via direct interaction with the receptors or epigenetic changes. Some documented disorders include decline in fertility, congenital malformation of gonads development, and increased incidence of reproductive diseases and cancer (Adeel et al., 2017; Rashtian et al., 2019).

The increasing worldwide concern about environmental exposure to estrogens has prompted programs to understand and improve their removal from WWTPs, which are the main source of environmental pollution with estrogens (Leech et al., 2009; Yu and Chu, 2009). Some research studies also addressed estrogens removal from drinking water treatment plants, constructed wetlands, sewer, manure, poultry litter and diary disposal systems (Yu et al., 2013).

Removal of steroid estrogens is challenging due to their recalcitrant sterane structure and low aqueous solubility, which were found to be as low as 30, 3.6, 441, and 116 mg/L, for E1, E2, E3, and EE2, respectively (Zhang et al., 2015). While WWTPs are essential for eliminating estrogens; they are not designed to eliminate them completely (López-Velázquez et al., 2021). Thus, estrogens are continuously discharged into surface water (López-Velázquez et al., 2025; Alfonso Murillo-Tovar et al., 2025). Various studies have evaluated estrogens removal from WWTPs using microfiltration, activated carbon adsorption, chemical degradation, and advanced treatment such as reverse osmosis. However, despite the relative efficiency of the physical and chemical techniques, biological methods, predominantly the use of biofilms, are emerging (Gadupudi et al., 2021).

Biological removal of estrogens in WWTPs includes deconjugation, complete degradation by heterotrophic microorganisms, or co-metabolism (Fernández et al., 2017; Ke et al., 2007; Liu et al., 2016; Zhang et al., 2024). However, the removal efficiency varies from one plant to another and depends on several factors such as operating conditions, geological location, estrogen concentration, and the biological system type (Yu et al., 2013). Estrogen elimination is more efficient in WWTPs equipped with biological treatment systems with long solid and hydraulic retention times. The latter enable longer contact time for better estrogen adsorption and sufficient time for the growth of slow-growing estrogen degraders (Maeng et al., 2013).

In all cases, it is generally acknowledged that estrogens are challenging substrates for microbial utilization due to their physicochemical properties such as low aqueous solubility, low number of functional groups, the presence of four alicyclic rings, and the presence of two quaternary carbon atoms (Olivera and Luengo, 2019), which may contribute to the various removal efficiencies reported in the literature, ranging between 14–94%, 76–92%, and 83–87% for E1, E2, and EE2, respectively (Baronti et al., 2000). It is also worth noting that steroid estrogens exhibit different susceptibilities to microbial degradation, following the descending order E3 > E2 > E1 > EE2 (Coello-Garcia et al., 2019).

Many studies addressed estrogen biodegradation in the environment, with more focus on WWTPs, which revealed a diversity of estrogen-degrading microorganisms and biodegradation/biotransformation mechanisms. Several factors may affect the type of estrogen metabolic mode in different bacteria. However, the key determinants appear to be the bacterial species and the applied growth conditions, which are discussed and analyzed in the following sections.

## 4 Microbial biodegradation and biotransformation of estrogens

### 4.1 Estrogen-degrading microorganisms

Microbial biodegradation was reported as the major mechanism which removes estrogens from engineered and natural polluted environments (Yu et al., 2013; Huang et al., 2014; Liyanage et al., 2024). Despite many studies on estrogens biodegradation, there are still many knowledge gaps regarding the catabolic pathways. Moreover, the involved enzymes, genes, and their regulation mechanisms are not fully characterized yet. Degradation of estrogens by aerobic bacteria has been reported, and many estrogens–degrading bacteria have been isolated from various environments (Yu et al., 2013; Zhang et al., 2015; Olivera and Luengo, 2019; Chiang et al., 2020; Pratush et al., 2020a).

Estrogens, like other steroids, are carbon-rich and highly reduced compounds that are abundant and ubiquitous in the environment. Therefore, they represent a precious carbon and energy source for some microorganisms (Chiang et al., 2020). In fact, some microorganisms (including bacteria, yeasts, fungi, and microalgae) can perform biotransformation reactions on estrogens (Pratush et al., 2020a). However, only dedicated bacteria have the ability to mineralize estrogens (complete degradation to CO_2_) (Bergstrand et al., 2016; Holert et al., 2018). Recent studies identified steroid-degrading bacteria and elucidated the biodegradation mechanisms using both culture-dependent and culture-independent approaches.

Bacteria that can degrade estrogens under aerobic conditions were isolated mainly from activated sludge, soil, compost, sandy aquifers, and very little from the marine environment. Although the isolates belong to different phyla, most estrogen-degrading bacteria reported to date belong mainly to the Actinomycetota (Actinobacteria) and Pseudomonadota (Proteobacteria) (Supplementary Tables S1, S2) (Bergstrand et al., 2016; Chiang et al., 2020; Pratush et al., 2020a; Shah et al., 2024).

Key enzymes involved in aerobic estrogen biodegradation and biotransformation are classified in different categories including dehydrogenases, hydroxylases, ring-cleavage dioxygenases, isomerases, monooxygenases, hydratases, and demethylases, as well as cytochrome P450 (Chiang et al., 2020; Pratush et al., 2020a).

### 4.2 Initial degradation and transformation reactions

**Figure 2:**
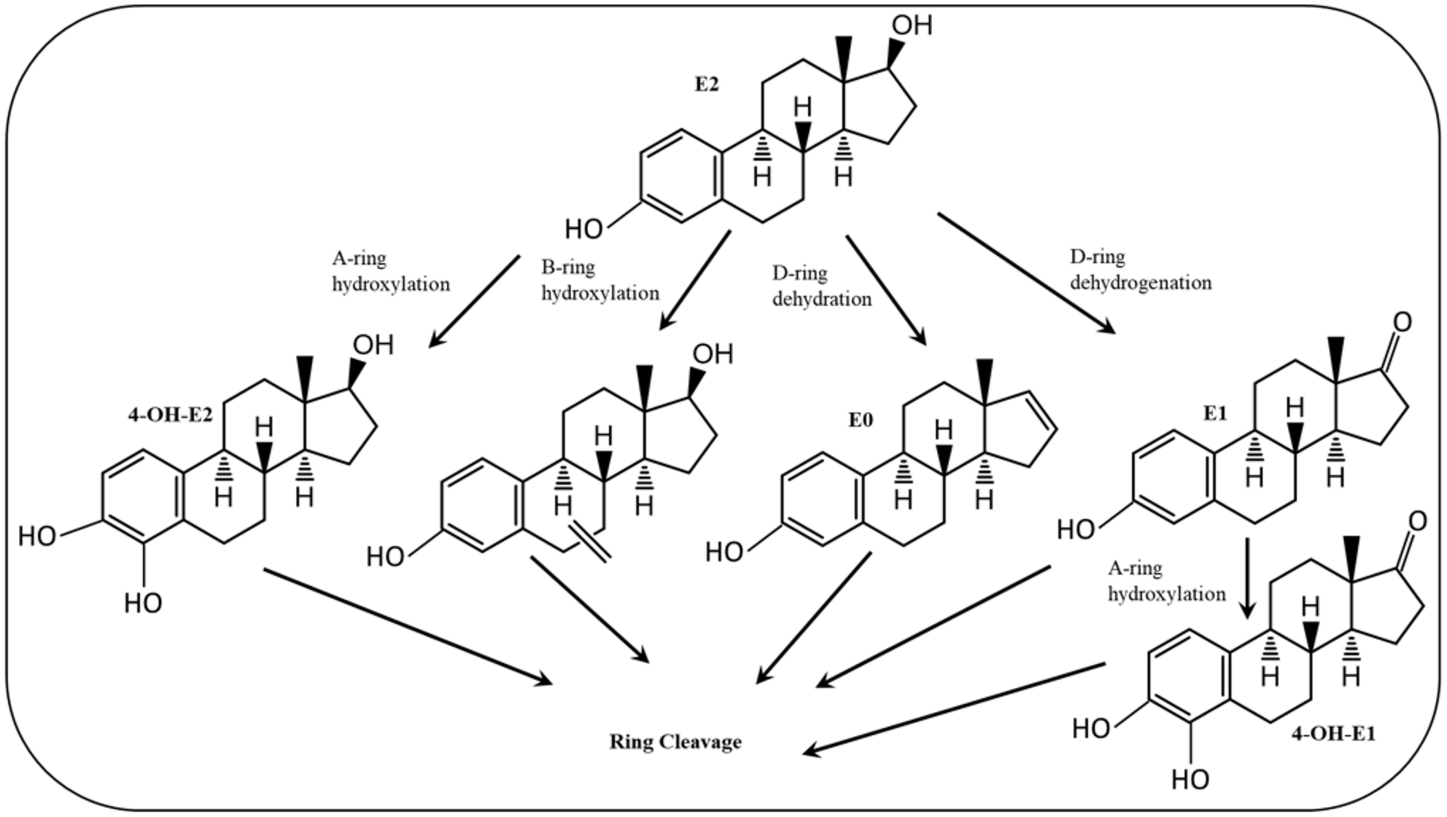
Initial reactions of major E2 degradation pathways (Mensitieri et al., 2023).

Various estrogen biotransformation reactions were reported, including hydroxylation, dehydrogenation, and ring cleavage reactions (Figure 2). Most of the studies have focused on the aerobic biodegradation E1 and E2 as model substrates. On the contrary, E3 and EE2 have not been less thoroughly investigated. Moreover, the biodegradation of estrogens under anaerobic conditions was reported only recently (Wang et al., 2020).

Despite the high structural similarity among estrogens, a diversity of biotransformation reactions and degradation pathways have been reported in the literature. Some of those reactions/pathways were proposed based on the detection and identification of metabolic intermediates. Another important feature of estrogen biodegradation is the fact that estrogens are interconvertible, i.e., one estrogen can be transformed to another during biodegradation or biotransformation. To date, three major catabolic pathways for steroid biodegradation have been reported. Under aerobic conditions, sterols and androgens are degraded via the 9,10-*seco* pathway, while estrogens are commonly degraded via the 4, 5-*seco* pathway.

**Figure 3:**
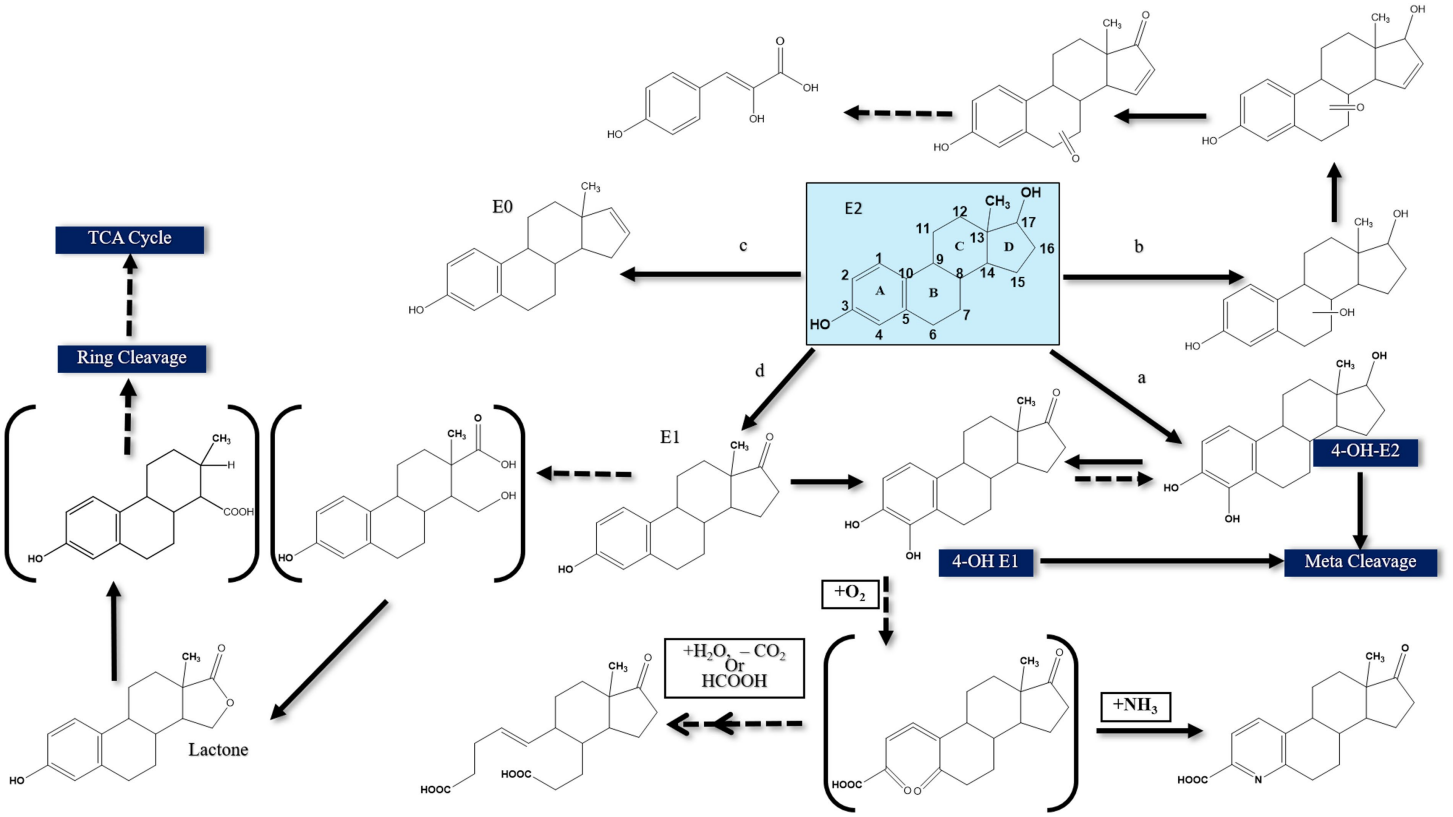
Proposed E2 degradation pathways by aerobic bacteria (Solid lines indicate confirmed pathways. Dashed lines indicate uncertain pathways) [modified from Yu et al., 2013].

On the contrary, under anaerobic conditions sterols and androgens are catabolized via the 2,3-*seco* pathway, while the anaerobic degradation of estrogens has not been studied in detail (Chiang et al., 2020; Jacoby et al., 2025). Regardless of the oxygen availability, the three pathways merge at a common key intermediate, namely HIP (3aα-H-4α(3′-propanoate)-7aβ-methylhexahydro-1,5-indanedione), which is subsequently degraded via a common central pathway to produce metabolites that are further degraded through central metabolic networks (Figure 3). In the following, different estrogen biodegradation and biotransformation mechanisms will be presented. For clarity, E2 and E1 will be discussed together because their degradation pathways are linked. Subsequently, EE2 and E3 degradation pathways will be presented separately.

### 4.3 Aerobic degradation of E2 and E1

The first E2-degrading bacterium was isolated from activated sludge and identified as *Novosphingobium tardaugens* sp. nov. (Fujii et al., 2002), and Coombre et al. (1966) proposed the first pathway for E1 degradation by the soil bacterium *Nocardia* sp. E110. It was proposed that a dioxygenase catalyzes the cleavage of the A-ring of E1 and this pathway was included as a part of E2 degradation pathways reported in subsequent studies, proposing that E2 degradation can be initiated by one of the following reactions: (a) hydroxylation of the A-ring at C4, (b) hydroxylation of a saturated ring, (c) dehydration of the D-ring at C17, and (d) dehydrogenation of the D-ring at C17 (Yu et al., 2013) (Figure 3).

The first mode of degradation was reported by Kurisu et al. (2010) who detected 4-OH-E2 during degradation by the soil isolate *Sphingomonas* sp. ED8, which suggests that E2 was hydroxylated at C4. The authors proposed that degradation of this hydroxylated product proceeds via *meta* cleavage. Kurisu et al. (2010) detected other products of E2 degradation by the ED8 strain, including hydroxy-E2, keto-E2, keto-E1, and 3-(4-hydroxyphenyl)-2-hydroxyprop-2-enoic acid. This latter product suggested that E2 degradation was initiated at a saturated ring. However, the authors did not further investigate how the saturated ring was cleaved. In another study, Nakai et al. (2011) used *Nitrosomonas europeae* to degrade E2 and detected a metabolite identified as estra-1, 3, 5 (10), 16 tetraen-3-ol (estratetra enol, or E0), which results from dehydration at C17 of the D-ring. *Nitrosomonas europeae* further degraded E0 to non-estrogenic compounds.

Transformation of E2 to E1 via dehydrogenation of C17 at the D-ring was also reported (Yu et al., 2007). Estrone can be further transformed to 4-OH-E1, followed by further degradation via *meta* cleavage (Kurisu et al., 2010). Lee and Liu (2002) suggested another degradation pathway for E2 when utilized by mixed sewage bacteria based on the detection of a new metabolite designated XI, which contains a lactone structure at the D-ring. All those previous studies proposed some biotransformation reactions, albeit complete pathway for estrogen degradation was not elucidated in terms of metabolic intermediates, genes, and enzymes, thus rendering the assessment of the fate and biodegradation potential of estrogens in the polluted environments challenging. Furthermore, the structural elucidations were mostly based on mass-spectrometry and no ring-cleave products were reported.

#### 4.3.1 Elucidation of the 4,5-*seco* pathway

**Figure 4:**
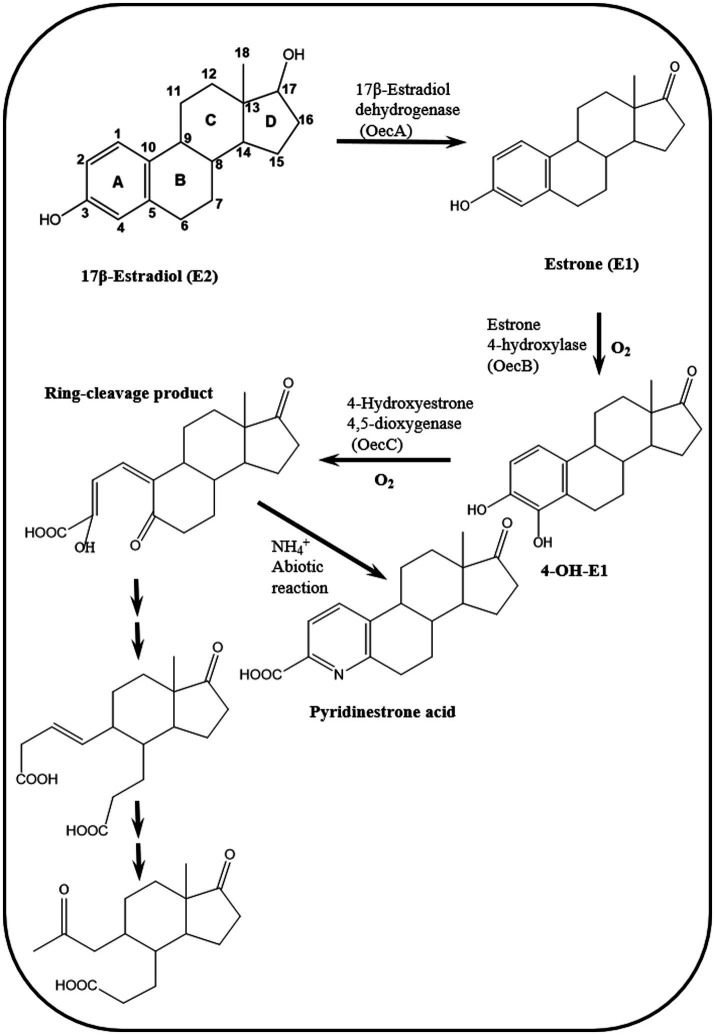
Reactions of the 4,5-*seco* pathway of aerobic biodegradation of estrogens in activated sludge and *Novosphingobium* sp. SLCC, showing intermediates downstream of the A-ring cleavage product (Chen et al., 2017, 2018).

To address these knowledge gaps, Chen et al. (2017) adopted functional genomic analyses and enzyme characterization to study E2 degradation by *Sphingomonas* sp. KC8. They showed that E2 degradation by the KC8 strain starts by oxidation of the C17 hydroxyl group leading to E1, followed by hydroxylation at C4 of the A-ring to produce 4-OH-E1. The A-ring is then opened via *meta* cleavage. These reactions constitute the initial steps of the so called 4,5-*seco* pathway for aerobic bacterial degradation of E2/E1 (Figure 4). A key feature of this pathway is the abiotic reaction of the A-ring cleavage product with NH_4_^+^ to produce pyridinestrone acid (PEA), a typical side product and functional marker of the 4,5-*seco* pathway. Further characterization of 17*β*-E2 dehydrogenase (OecA), which catalyzes the transformation of E2 to E1, and 4-OH-E1 4,5-dioxygenase, which catalyzes the *meta* cleavage of the A-ring, was conducted by Chen et al. (2017). Moreover, the genes encoding these two enzymes were identified in the genome of the KC8 strain.

Two gene clusters were specifically expressed during growth on E2 compared to cultures grown on testosterone. In one of these gene clusters, Chen et al. (2017) identified a gene (*oecB*), proposed to encode a putative flavin-dependent monooxygenase, which catalyzes hydroxylation of E1 at C4 of the A-ring. However, this enzyme was not further characterized. Enzymes encoded by gene cluster II were proposed to catalyze the A/B rings degradation. The authors also identified gene cluster III, which was similarly expressed in E2- and testosterone-grown cells. The gene products of this gene cluster exhibit high identity to β-oxidation enzymes and were proposed to be involved in the degradation of the C/D-rings of E2. Detection of PEA in [3,4C-^13^C]E1-spiked river water and activated sludge samples from a WWTP suggested that the 4,5-*seco* pathway might play a role in estrogen degradation in the environment (Chen et al., 2017).

Further biochemical and microbiological studies were performed by Chen et al. (2018) to detect additional downstream intermediates of the 4,5-*seco* pathway and to assess the ecological relevance of estrogen-degrading bacteria. In activated sludge samples incubated with El, PEA and two A/B-rings cleavage products were detected (Figure 4). Furthermore, PCR detected homologs of the alphaproteobacterial *oecC*, a functional marker of the 4,5-*seco* pathway. Metagenomics analysis revealed that *Novosphingobium* spp. are key estrogen degraders in activated sludge, which was corroborated by the isolation of *Novosphingobium* sp. SLCC strain from the E1-amended activated sludge. In batch cultures, this strain degraded E1 and produced the A/B-rings cleavage products detected in the activated sludge, thus confirming their role in E1 degradation.

**Figure 5:**
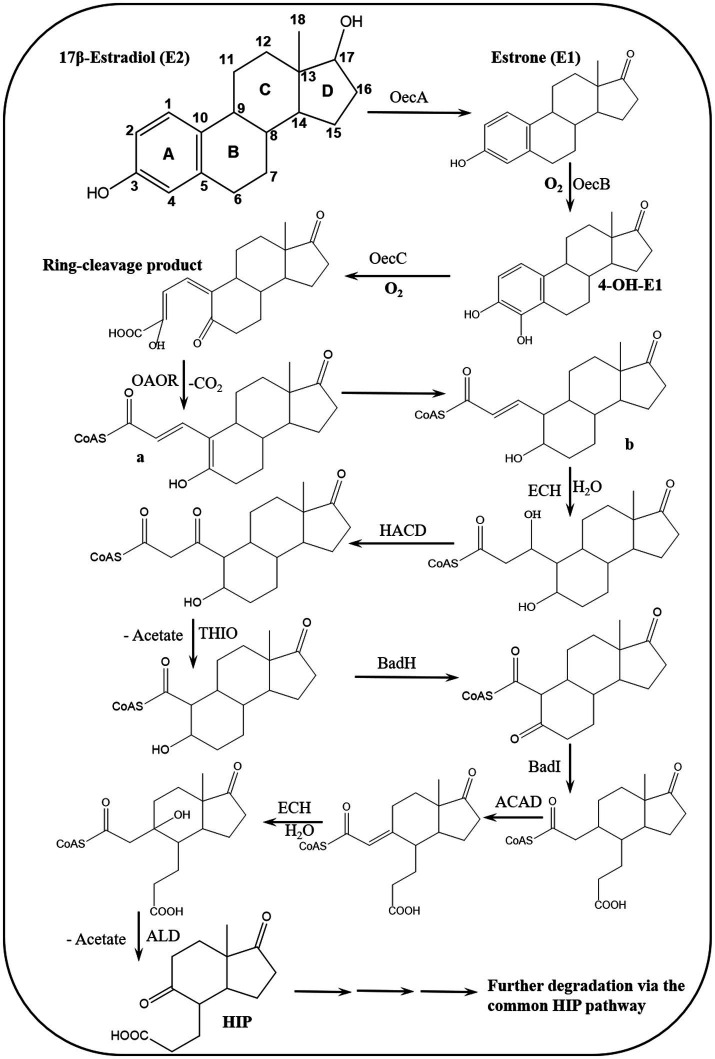
Proposed bacterial degradation pathway of natural estrogens. ACAD, acyl-CoA dehydrogenase; ALD, aldolase; BadH, 2-hydroxycyclohexanecarboxyl-CoA dehydrogenase; BadI, 2-ketocyclohexanecarboxyl-CoA hydrolase; ECH, enoyl-CoA hydratase; HACD, β-hydroxyacyl-CoA dehydrogenase; OAOR, 2-oxoacid oxidoreductase; THIO, thiolase (Wu et al., 2019).

Further investigations on the 4,5-*seco* pathway were reported by Wu et al. (2019) using *Sphingomonas* sp. KC8 as a model organism and applying NMR and UPLC-HRMS (Ultraperformance Liquid Chromatography-High Resolution Mass Spectroscopy) to identify metabolic intermediates of E1 degradation. In E1 cultures, the authors could detect and identify 4-norestrogen-5(10)-en-3-oyl-coenzyme A (CoA) and the corresponding non-conjugate (4-norestrogenic acid), in addition to the common metabolite of microbial steroid metabolism (HIP). Based on these findings, they proposed that after A-ring cleavage, C4 is removed by oxidative decarboxylation by a 2-oxoacid oxidoreductase to produce 4-norestrogen-5(10)-en-3-oyl-CoA. Then the B-ring is opened by hydrolysis and β-oxidation reactions transform the A/B-ring cleavage product into the common metabolite HIP (Table 3; Figure 5).

**Table 3:** Key enzymes of bacterial aerobic estrogen degradation via the 4,5-*seco* pathway and the HIP pathway.

Pseudomonadota (*Sphingomonas* sp. KC8, *Novosphingobium tardaugens* NBRC 16725, *Croceicoccus estronivorus*)	Actinomycetota (*Rhodococcus* sp. B50, *Rhodococcus hoagi* DSSKP-R-001, *Dietza* sp. B32, *Mycobacterium tuberculosis*, *Mycobacteroides chelonae* S00154)	Catalytic role
17β-E2 Dehydrogenase (OecA)	17β-Hydroxysteroid (short-chain) dehydrogenase	Transformation of E2 to E1
Flavin-dependent monooxygenase (OecB)	P450 E1-4-Hydroxylase (AedA)	Hydroxylation of the A-ring of E1
4-OH-E1 4,5-Dioxygenase (OecC)	4-OH-E1 4,5-Dioxygenase (AedB)	Cleavage of the A-ring of 4-OH-E1
2-oxoacid-ferredoxin Oxidoreductase (EdcC)	2-Hydroxyacid dehydrogenase (AedG), decarboxylase (AedH) and a CoA-ligase (AedJ).	Degradation of the A/B rings (decarboxylation of the meta-cleavage product and thioesterification of the resulting metabolite with CoA)
Reductase	Acyl-CoA dehydrogenase (AedP)	Degradation of the A/B rings
Enoyl-CoA hydratase	Enoyl-CoA hydratase (AedD)	Degradation of the A/B rings
3-Hydroxyacyl-CoA dehydrogenase	3-Hydroxyacyl-CoA dehydrogenase (AedE)	Degradation of the A/B rings
Thiolase	Thiolase (AedK, AedF)	Degradation of the A/B rings
2-Hydoxycyclohexanecarboxyl-CoA dehydrogenase	A putative medium-chain length fatty acid-CoA ligase	Degradation of the A/B rings
2-Ketocyclohexanecarboxyl-CoA hydrolase	Acyl-CoA dehydrogenase (AedN)	Degradation of the A/B rings
Acyl-CoA dehydrogenase	3-Hydroxyacyl-CoA dehydrogenase, (AedG)	Degradation of the A/B rings
Enoyl-CoA hydratase	Enoyl-CoA hydratase (AedM)	Degradation of the A/B rings
Aldolase	Enoyl-CoA hydratase (AedO)	Degradation of the A/B rings
CoA acyltransferase	Short-chain-type dehydrogenase/reductase	Degradation of the C/D rings
MaoC dehydratase	HIEC-CoA hydrolase	Degradation of the C/D rings
Short-chain dehydrogenase/reductase	COCHEA-CoA hydrolase	Degradation of the C/D rings
Acyl-CoA dehydrogenase	5-OH HIC-CoA reductase	Degradation of the C/D rings
Short-chain oxidoreductase	β-Keto CoA thiolase	Degradation of the C/D rings
acetyl-CoA acetyltransferase	5-Oxo HIC-CoA oxidase	Degradation of the C/D rings
Enoyl-CoA hydratase	Acyl-CoA dehydrogenase	Degradation of the C/D rings
Monooxygenase	HIP-CoA synthetase	Degradation of the C/D rings
Lipid-transfer protein	Acyl-CoA dehydrogenase	Degradation of the C/D rings
Thiolase	MOODA-CoA dehydrogenase	Degradation of the C/D rings
CoA transferase	Acyl-CoA dehydrogenase	Degradation of the C/D rings

Names of the enzymes and their proposed or validated catalytic role were retrieved from Casabon et al. (2013), Crowe et al. (2017), and Olivera and Luengo (2019). HIEC-CoA, (7aS)-7a-Methyl-1,5-dioxo-2,3,5,6,7,7a-hexahydro-1H-indene-4-carboxyl-CoA; COCHEA-CoA, (R)-2-(2-carboxyethyl)-3-methyl-6-oxocyclohex-1-ene-1-carboxyl-CoA; 5-OH HIC-CoA, 3aα-H-4α(carboxyl-CoA)-5-hydroxy-7aβ-methylhexahydro-1-indanone; MOODA-CoA; 4-methyl-5-oxo-octanedioyl-CoA.

Although PEA was commonly regarded as a dead-end product, it may undergo further reactions such as ring cleavage as reported recently in *Microbacterium proteolyticum* ZJSK01 (Chen et al., 2025). Furthermore, E2 degradation can be initiated at the saturated rings after hydroxylation of the A-ring to produce 4-OH-E2 (Chen et al., 2025). Consolidated cleavage of both the A- and B-ring of E2 after transformation to 4-OH-E2 was enhanced in the presence of Fe(III) which promoted the expression of the involved monooxygenase and extradiol dioxygenase (Li et al., 2024).

A diversity of estrogen biotransformation reactions was reported by Zhu et al. (2024), who identified 18 transformation products of E2 in a culture medium inoculated with the human intestinal fungus *Aspergillus niger* RG13B1. Hydroxylation, oxidation, dehydrogenation, and alkylation were the key transformation reactions. While hydroxylation reactions were the most common and occurred at various positions on the E2 structure, oxidation and alkylation reactions were mostly on the D- and B-rings. Notably, no ring-cleavage products were detected, further confirming that the metabolic mode is biotransformation not complete degradation.

Although the majority of research reported on E1 and E2 degradation focused on members of the Pseudomonadota and Actinomycetota, less information is available regarding the involved genes, enzymes and pathways for actinobacterial estrogen degraders, and Hsiao et al. performed a series of detailed studies to fill these knowledge gaps. In the first study, Hsiao et al. (2021) applied metabolomics and gene disruption studies using a soil isolate *Rhodococcus* sp. B50 as a model estrogen-degrading strain which utilized E1, E2, E3, testosterone, or cholesterol as a sole carbon and energy source. In E1-fed resting cell assays, UPLC-APCI-HRMS analysis revealed key metabolites of the 4,5-*seco* pathway, such as PEA and HIP.

Comparative genomic analysis revealed a putative estrogen degradation gene cluster carried on a mega plasmid and encodes a cytochrome P450 E1-4-hydroxylase (*aedA*) and a *meta* cleavage dioxygenase (*aedB*) involved in estrogen degradation via the 4,5-*seco* pathway. The function of these two enzymes was validated via gene disruption and phenotype analysis, showing functional similarity with the corresponding homologs from Pseudomonadota, EdcA and EdcB (Ibero et al., 2020) and OecA and OecB (Chen et al., 2017).

Despite this functional similarity, the amino acid sequence identity between the actinobacterial and proteobacterial homologs is relatively low (<40%). Subsequently, the authors used the extracellular metabolites PEA and HIP in addition to primers specific to the actinobacterial 4-hydroxyestrone 4,5-dioxygenase gene (*aedB*) as functional biomarkers to study estrogen biodegradation in urban estuarine sediments. Using E1-spiked mesocosms and PCR-based functional assays, it was concluded that estrogen biodegradation in the estuarine sediments proceeds via the 4,5-*seco* pathway, and Actinomycetota are active estrogen degraders in this ecosystem.

In a follow up study, Hsiao et al. (2022) applied comparative transcriptomics, gene disruption, and metabolite analysis to unravel genes and intermediates of downstream degradation of the estrogen A/B rings using *Rhodococcus* sp. B50 as a model. Analysis of the transcriptomes of B50 cultures grown on E1 compared to control cultures on cholesterol or testosterone showed that genes involved in C/D-rings degradation were similarly expressed in all three cultures. On the contrary, the *aed* gene cluster, which was identified by Hsiao et al. (2021) was significantly upregulated in the E1-grown culture. This gene cluster carries genes encoding AedA (E1 hydroxylase), AedB (*meta* cleavage dioxygenase), a putative medium-chain length fatty acid-CoA ligase, genes encoding two sets of *β*-oxidation enzymes including acyl-CoA dehydrogenase (AedN, AedP), enoyl-CoA hydratase (AedD, AedM, AedO), 3-hydroxyacyl-CoA dehydrogenase, (AedE, AedG) and thiolase (AedF, AedK). The authors then constructed knockouts for the genes AedF and AedK and validated their function via phenotype and metabolite analysis. Based on their findings, an estrogen degradation pathway for Actinomycetota was established. In addition to the common metabolites of the 4,5-*seco* pathway, three novel metabolites were identified including 4,5-*seco*-estrogenic acid, 5-oxo-4-norestogenic acid, 2, 3, 4-trinorestrogenic acid.

Obviously, estrogen degradation in Actinomycetota proceeds via the 4, 5-*seco* pathway as reported for Pseudomonadota. However, there are still some differences that distinguish both bacterial groups. For instance, actinobacterial estrogen metabolites bear a 5-oxo group, whereas the corresponding metabolites in Pseudomonadota have a hydroxy group at C5. Moreover, the ring-A cleavage products in Pseudomonadota possess 3 unsaturated bonds (at C1, C3, and C5), while in Actinomycetota the corresponding 4,5-*seco*-estrogenic acid has only two double bonds (at C1 and C5) and is less likely recyclized. Oxidation of the A-ring-cleaved products also reveals some differences. In *Sphingomonas* sp. KC8, oxidative decarboxylation after opening of the A-ring is catalyzed by a 2-oxoacid oxidoreductase (EdcC), a member of the indolepyruvate ferredoxin oxidoreductase family, to produce 4-norestrogen-5 (10)-en-3-oyl-CoA (Wu et al., 2019; Ibero et al., 2020). In *Rhodococcus* sp. B50, an EdcC homolog is lacking in the genome. Hence, the EdcC function may be carried out by a putative 2-hydroxyacid dehydrogenase (AedG), a decarboxylase (AedH) and a CoA-ligase (AedJ).

**Figure 6:**
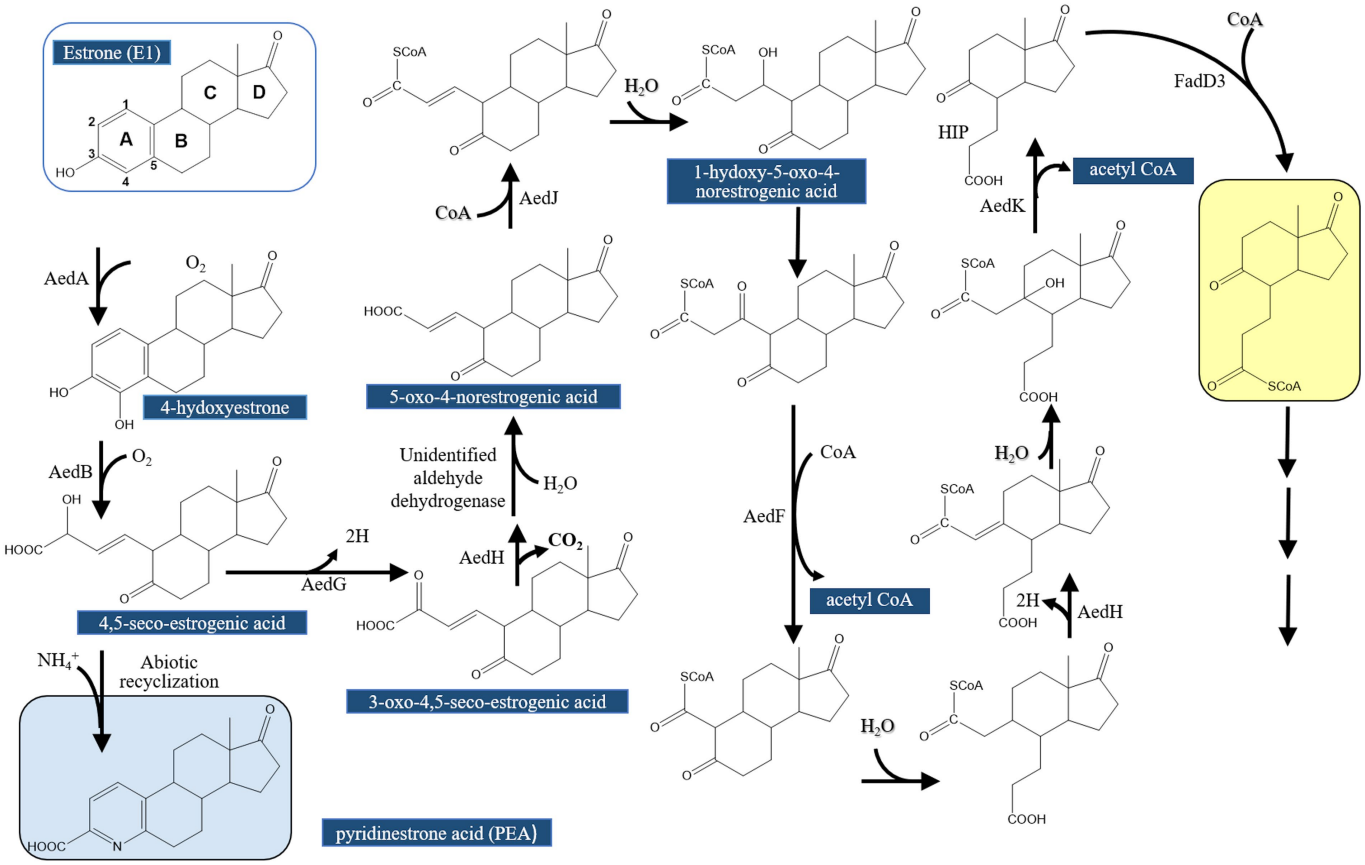
Proposed estrogen degradation pathway in Actinobacteria (Actinomycetota). AedG, AedH, and AedJ were functionally characterized in this study [modified from Hsiao et al., 2023].

Hsiao et al. (2023) continued their investigations on the topic and conducted further comparative genomics, gene disruption, and metabolite analysis studies to validate the proposed function for the actinobacterial enzymes AedGHJ. They first revealed that the genes *aedGHJ* are found exclusively in the genomes of estrogen-degrading Actinomycetota. In contrast, the *α*-oxoacid-ferredoxin oxidoreductase EdcC is unique to estrogen-degrading Pseudomonadota. These findings suggested the presence of phylum-specific functional markers that delineate estrogen degradation mechanisms. Gene disruption experiments and metabolite analysis validated the proposed functions for AedGHJ proteins (Table 3; Figure 6). The authors further detected a C_17_ metabolite (1-hydroxy-5-oxo-4-norestrogenic acid) that accumulated in the wild type.

**Figure 7:**
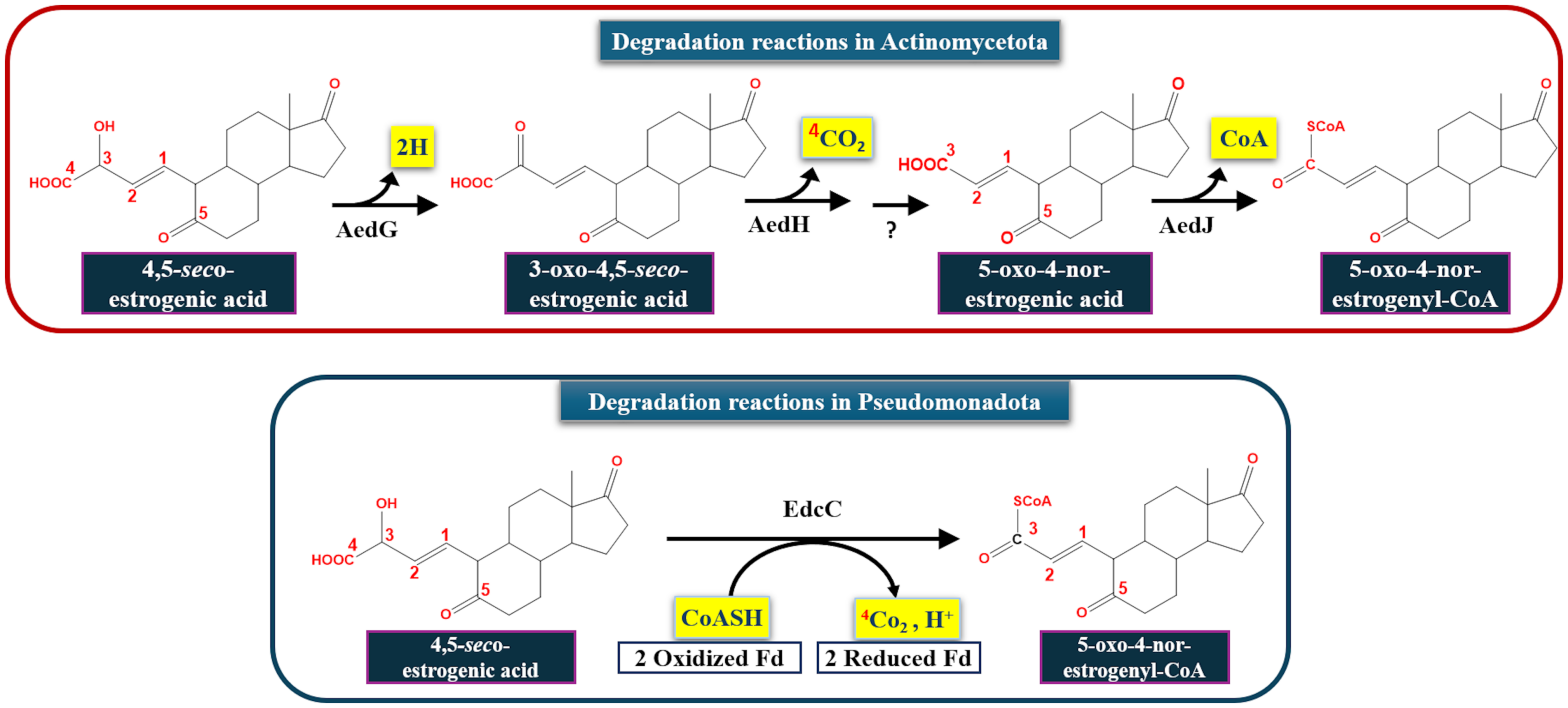
Bacterial taxa-specific enzymes involved in aerobic estrogen degradation reactions. Fd, ferredoxin (Hsiao et al., 2023).

Based on these findings, it appears that activation and degradation of the estrogenic A-ring proceeds via different strategies. In Pseudomonadota, a single oxidative decarboxylation reaction catalyzed by an α-oxoacid-ferredoxin oxidoreductase EdcC removes the C4 of E1 and produces a CoA-ester C_17_ metabolite (Ibero et al., 2020; Wu et al., 2019). Homologs of EdcC are lacking in estrogen-degrading members of the Actinomycetota, which depend on the enzymes AedGHJ to degrade the A-ring. Accordingly, these phylum-specific genes were used as biomarkers to study estrogen degradation in the environment (Table 3; Figure 7) using degenerate specific primers for the *aedJ* gene and *edcC* genes and phylum-specific 16S rRNA gene primers.

Quantitative PCR assays using DNA from various estrogen-contaminated ecosystems in Taiwan revealed that *γ-Proteobacteria* were most abundant in the examined environmental samples. However, the actinobacterial gene *aedJ* was more abundant than the proteobacterial gene *edcC* in most of the examined samples, particularly those collected from aquatic ecosystems, suggesting that the *aedJ* gene can be adopted as a specific biomarker to probe for estrogen-degrading Actinomycetota in the environment due to the following reasons: (a) the biochemical function of the AedJ enzyme was characterized and its substrate and product were identified, (b) an *aedJ* homolog is lacking in estrogen-degrading Pseudomonadota and non-estrogen-degrading Actinomycetota, (c) the long sequence of *aedJ* (1,500 bp) enables the design of specific primers, and (d) *aedJ* is highly expressed only in the presence of estrogens (Hsiao et al., 2022). Similarly, the gene encoding α-oxoacid-ferredoxin oxidoreductase *edcC* can be used as a specific biomarker for estrogen-degrading Pseudomonadota.

While estrogen degradation via the 4,5-*seco* pathway by other *Rhodococcus* spp. was proposed (Tian et al., 2022), it appears that alternative pathways could exist in different actinobacterial members. This could be inferred from a study on a mangrove sediment isolate, *Rhodococcus* spp., which utilized E1 as the sole carbon and energy source and produced two non-accumulating E1 metabolites, namely, 3-hydroxyandrosta-5,7,9(11)-trien-17-one and androsta-1,4,6-triene-3,17-dione (Pratush et al., 2020b). Genomic analysis revealed that the strain BH2-1 has 46 genes belonging to 6 major steroid degradation gene classes including cholesterol oxidase, steroid *Δ*-isomerase, cytochrome P450 monooxygenase, 3δ-steroid-1 dehydrogenase, 3-steroid-9α-hydroxylase, and 3α,20β-hydroxysteroid dehydrogenase.

Based on LC-MS analysis, E1, E3, and dehydroepiandrosterone, androsta-1,4-diene-3,17-dione, homovanillic acid, and vanillylmandelic acid, were detected as E2 metabolites in *Microbacterium resistens* MZT7, isolated from farm-derived activated sludge (Hao et al., 2022). Transformation of E2 to E1 in *Microbacterium* sp. MZT7 is catalyzed by a 17*β*-hydroxysteroid (short-chain) dehydrogenase which is active even in synthetic livestock wastewater (Hao et al., 2023). A similar study identified E1, E3, and dehydroepiandrosterone, androsta-1,4-diene-3,17-dione, etiocholanolone, 2-methoxyestradiol, homovanillic acid, and 3,4-dehydroxyphenylpropionic acid as E2 metabolites of *Rhodococcus* sp. RCBS9 strain isolated from soil of a dairy farm (Hao et al., 2024). Accordingly, several potential E2 degradation pathways were proposed by Hao et al. (2022, 2024), although no quantification data was reported to properly assess the relative significance of the different proposed pathways. The physiological or ecological significance of having several biodegradation pathways for the same compound was not discussed. Moreover, the possibility that some of the detected metabolites could result from unspecific biotransformation reactions was not highlighted. In those studies, the detection of androgenic metabolites is of particular interest because conversion of estrogens into androgens is thermodynamically challenging and has only been reported recently as a key step in anaerobic estrogen degradation (Wang et al., 2020), thus contrasting with the findings of Hao et al. (2022, 2024), which were reported for aerobic E2 degradation. It was not reported in those studies whether the genomes of the studied bacteria (*Rhodococcus* sp. RCBS9 and *Microbacterium* sp. MZT7) carry genes encoding homologs of the cobalamin-dependent methyltransferases which catalyze the estrogen retroconversion to androgens (Wang et al., 2020).

*Gordonia* sp. strain R9, isolated from an enrichment culture of chicken leachate, degraded E2, E1, E3, and EE2 as a sole carbon and energy source. E2 degradation by the strain R9 produced several metabolic intermediates including E1, E3, (3Z)-3-(3-hydroxy-3a-methyl-7-oxododecahydro-6H-cyclopenta[a]naphthalene-6-ylidene) propanoic acid, and 3-hydroxy-3a-methyl-7-oxododecahydro-1H-cyclopenta[a]naphthalene-6-carboxylic acid. The latter two metabolites suggest that E2 degradation proceeds via cleavage of the A-ring. The authors also reasoned that detection of E1 and E3 in the E2-degrading culture may indicate the presence of alternative estrogen degradation pathways in the R9 strain (Liu et al., 2020). It is worth noting that no CoA-bound intermediates were reported in this study, in contrast to the studies of Wu et al. (2019) which could be differences in degradation mechanisms by actinobacterial and proteobacterial members.

Estradiol biodegradation was studied by Li et al. (2020) using a *Novosphingobium* sp. ES2-1 that was isolated from activated sludge in a domestic sewage treatment plant. Using ^13^C labeling, NMR and HRMS, and as reported by Chen et al. (2017, 2018) and Wu et al. (2019), the authors proposed that E2 is transformed to E1 then to 4-OH-E1 where the A-ring is opened, and PEA is produced. However, ring cleavage appeared to occur by successive monooxygenation reactions leading to long-chain ketonic structures. The authors also observed the production of several pyridine derivatives.

Degradation of E2/E1 via the 4,5-*seco* pathway was also reported in *Novosphingobium tardaugens* NBRC 16725 (Ibero et al., 2020). Transcript analysis in this bacterium revealed a gene cluster, designated *edc*, that encodes estrogen degradation proteins. *Comamonas testosteroni* JLU460ET is another Proteobacterium in which E2 was proposed to be degraded via the 4, 5-*seco* pathway based on the transcriptomic data. This was shown by Wang et al. (2023) via comparative transcriptomics, which revealed a 100 kb steroid degradation gene cluster including genes for sensing substrates in the environment, steroid uptake, ring-cleavage, and β-oxidation enzymes. However, no degradation metabolites were reported from E2-grown cultures.

#### 4.3.2 Alternative E1 and E2 degradation pathways

Some other estrogen degradation pathways, which are different from the 4,5-*seco* pathway, were reported in cyanobacteria, yeast, and Gram-positive bacteria, further attesting taxon-dependent variations in biodegradation pathways. However, those pathways were proposed solely based on metabolite detection. For instance, Sami et al. (2023) studied E1 biodegradation by the cyanobacterium *Spirulina* CPCC-695 and showed that it utilizes E1 as a carbon and energy source for growth without addition of co-substrates. The degradation intermediates included fatty alcohols, alkanes, and a degradation pathway was proposed which starts with the reduction of E1 to E2. Detection of these intermediates suggested E1 metabolism may include hydrogenation, dehydrogenation, oxidation–reduction, hydroxylation, and side chain cleavage reactions. Moreover, E1 metabolism was proposed to start by reduction of the C17 keto group to produce E2, and the D-ring is cleaved first after formation of E1-formate and E1-propionate. After ring opening, esterification, methylation, and hydrogenation reactions produce diones/diols such as 3-phenylundecane, cyclo-tetradecane, and tetradecane.

After methylation, 4, 6-dimethyldodecane undergoes dehydrogenation to form lauryl alcohol. Further degradation via oxidation reactions produces lauric acid that condenses with CoA. The resulting fatty acyl-CoA is further degraded to acetyl-CoA (Sami et al., 2023). This proposed pathway is completely different from the commonly reported 4, 5-*seco* pathway and does not converge at HIP. Although these findings suggest the presence of different estrogen degradation pathways in cyanobacteria, the authors did not present biochemical evidence at the enzyme level. Furthermore, the culture medium contained several other carbon sources in addition to E1, such as acetone (the solvent of E1), citric acid, ferric ammonium citrate, Na-EDTA, and Na-CO_3_, which questions the origin of at least some of the detected intermediates.

**Figure 8:**
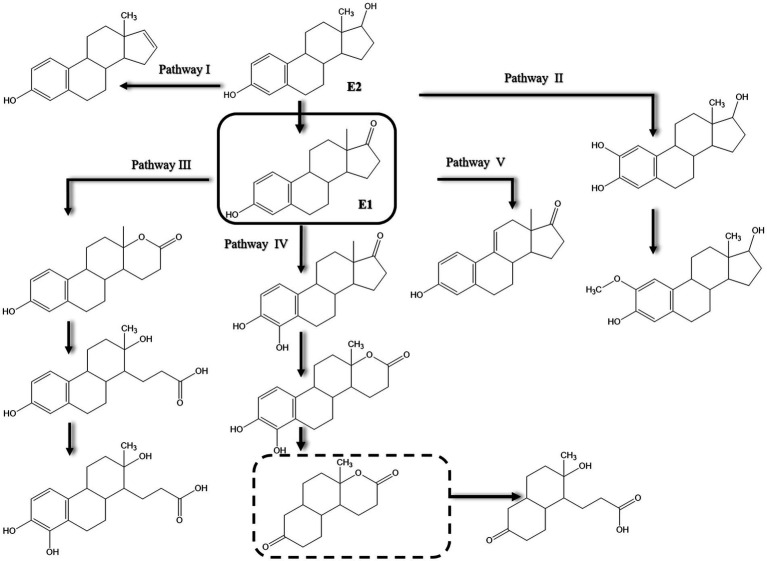
Proposed pathways of E2 biodegradation in *Candida utilis* CU-2 and *Lactobacillus casei* LC-1. The metabolite within the dashed rectangle was not detected by liquid chromatography–tandem mass spectrometry (LC-MS/MS) [modified from Ge et al., 2021].

Various pathways were proposed for E2 degradation by *Candida utilis* CU-2 and *Lactobacillus casei* LC-1 in the presence of additional carbon sources (Ge et al., 2021). Using LC-MS/MS analysis, 12 degradation products were detected in E2 biodegradation experiments and accordingly proposed five degradation pathways (Figure 8). In pathway I, degradation proceeds via a dehydration reaction at C17 of the D-ring to produce E0 as proposed earlier by Nakai et al. (2011). In pathway II, E2 is hydroxylated at the D-ring and then methylated. No ring cleavage products were reported for pathways I and II. As reported by several studies, E2 is first transformed to E1 in the other three alternative pathways reported by Ge et al. (2021), and E1 is then oxidized at the C-ring via dehydrogenation, or at the D-ring via oxygen incorporation as reported by Wang et al. (2019b), or at C4 of the A-ring to produce 4-OH-E1. However, further degradation of the intermediate proceeds via opening of the D-ring. Enzymes that could catalyze the proposed hydroxylation, methylation, and dehydrogenation reactions were not reported in this study.

#### 4.3.3 Estrogen degradation by bacterial consortia

Despite the known advantages of applying microbial consortia for degradation of environmental pollutants (Zhang and Zhang, 2022), degradation of estrogens by bacterial consortia has not been often reported. Mixed bacterial cultures were isolated from activated sludge and degraded estrogen in a minimal medium, however, the degradation pathways were not elucidated (Weber et al., 2005; Muller et al., 2010a). Estrogen degradation can be more efficient by mixed cultures compared to axenic cultures as shown by Wang et al. (2019a) for E2 degradation by a mixed culture of *Rhodococcus equi* DSSKP-R-001 and *Comamonas testosteroni* QYYZ0150409. Moreover, concerted metabolic capabilities of members of bacterial consortia could lead to novel biotransformations and/or degradation pathways that are lacking in pure-estrogen degrading bacteria. This was also shown by Wang et al. (2019a) who identified three unique E2 metabolites in the co-culture of *Rhodococcus equi* DSSKP-R-001 and *Comamonas testosteroni* QYYZ0150409 that were lacking in the pure cultures of these bacteria. These findings suggested E2 degradation via alternative pathways, all of which start by transformation of E2 to El. In one of the proposed pathways E1 is transformed to 4-hydroxyestrone, which is further hydroxylated at C-16 to give a metabolite that is further degraded via the TCA cycle. In the second pathway, the A-ring of 4-hydroxyestrone is cleaved between C-4 and C-5 and C-5 is oxidized to a carboxyl group, and the C-9–C-10 bond is then cleaved. In the third pathway, the B-ring of E1 is first cleaved by hydroxylation at C-9*α*, followed by hydroxylation of the A-ring at C-4 and cleavage between C-4 and C-5. Eventually, the C-5–C-6 bond is cleaved. However, neither pyridine derivatives or CoA thioesters were reported, and the involved enzymes and genes were not identified.

Estrone biodegradation was studied in E1-spiked activated sludge microcosms from a major WWTP in Bahrain, and the key metabolites of the 4,5-*seco* pathway, namely, 4-OH-E1 and PEA were detected, indicating that E1 was degraded via the 4,5-*seco* pathway (AlDhafiri et al., 2022). The alphaproteobacterial genera *Novosphingobium* and *Sphingoaurantiacus* were significantly enriched in the E1-spiked microcosms after 2 days, and the authors enriched a mixed culture from the activated sludge which degraded E1 in batch cultures. From this consortium, an alphaproteobacterial strain, identified as *Sphingobium estrogenivorans*, was isolated and degraded E1 in batch cultures, revealing the role of Alphaproteobacteria in E1 degradation.

### 4.4 Aerobic EE2 degradation

Biodegradation of EE2 has been less frequently reported, and most of the available studies were conducted with microbial cultures that can utilize/transform EE2 in the presence of co-substrates, thus following a cometabolic mode of degradation (O’Grady et al., 2009; Pauwels et al., 2008). This could be attributed to recalcitrance of EE2 compared to the natural estrogens (Zhang et al., 2015; Olivera and Luengo, 2019). Co-substrates may strengthen central metabolism, promote cell growth, and supply reducing power, and can even induce the expression of some enzymes that play a role in EE2 metabolism (Gröning et al., 2007; Tran et al., 2013; Fischer and Majewsky, 2014). Currently, no complete EE2 biodegradation pathway is available and knowledge of the involved genes, enzymes are largely lacking.

Only a few bacteria can degrade EE2 as a sole carbon and energy source such as *Sphingobacterium* sp. JCR5 (Haiyan et al., 2007) and *Rhodococcus equi* (Yoshimoto et al., 2004). The number of reported microorganisms that can transform or cometabolize EE2 is very limited including *Sphingomonas* spp., *Pseudomonas* spp., *Nitrosomonas europaea*, the cyanobacterium *Microcystis novacekii*, the microalgal strain *Selenastrum capricornutum*, and the fungi *Phoma* sp. and *Pleurotus ostreatus* (Flint, 2019; Pratush et al., 2020a). Degradation of EE2 occurs under aerobic and anaerobic conditions, albeit higher degradation rates were reported under aerobic conditions (Aris et al., 2014; Cajthaml et al., 2009). EE2 degradation was slow in marine sediments (Ying and Kookana, 2003) and aquifer material (Ying et al., 2003), while in agricultural soils (Colucci and Topp, 2001) and nitrifying activated sludge (Vader et al., 2000) it was more rapidly degraded. Earlier studies reported biotransformation of EE2 by some bacteria and microalgae (Haiyan et al., 2007; Della Greca et al., 2008) including *Sphingobium* sp. JCR5, *Rhodococcus zopfii*, and *Nitrosomonas europaea* EE2 (Shi et al., 2002; Yoshimoto et al., 2004; Haiyan et al., 2007; Aris et al., 2014). One of the possible degradation pathways starts by transforming EE2 to E1, then E3, but no further degradation was reported (Li et al., 2013a, 2013b), or transformation of EE2 to E1, followed by ring cleavage (Cajthaml et al., 2009). At low concentrations, EE2 could be co-metabolized with E1, E2, or E3 in sludge and wastewater; however, no further metabolites were detected (Cajthaml et al., 2009; Writer et al., 2012; Rhee et al., 2014).

**Figure 9:**
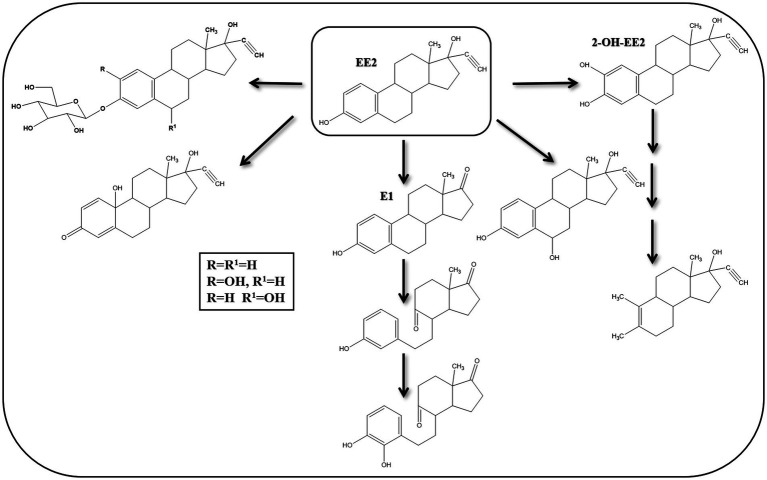
Proposed EE2 degradation pathways in bacteria and algae [modified from Yu et al. (2013)].

Several studies reported biotransformation reactions for EE2 without proposing complete degradation pathways (Figure 9). Yi and Harper (2007) detected 2-OH-EE2 during their studies on EE2 degradation by a nitrifying culture and showed that the A-ring was cleaved before the other rings, probably due to the higher electron density of the A-ring carbon units. These findings suggested that EE2 can be cometabolically transformed under nitrification conditions.

Some algal species can transform EE2 into EE2-glucoside, 3-*β*-D-glucopyranosyl-2-hydroxy-EE2, 3-β-D-glucopyranoside-6β-hydroxy-EE2, 17α-ethinyl-1,4, estradien-10,17β-diol-3-one, and 6-α-OH-EE2 (Della Greca et al., 2008). Thus, hydroxylation, oxidation, and glycosylation reactions were the main biotransformation reactions. In another study, Haiyan et al. (2007) isolated *Sphingobacterium* sp. JCR5 strain from activated sludge and showed that it can grow on EE2 as a sole carbon and energy source. Mass spectrometry analysis revealed that EE2 is first transformed to E1, followed by hydroxylation and oxidation reactions at C9, leading to B-ring cleavage. The A-ring is then hydroxylated at C4.

Co-metabolism of EE2 was studied with several bacteria isolated from compost. The isolated bacteria degraded E1, E2, and E3. However, EE2 was not degraded when used as a sole carbon source, albeit it was degraded in the presence of E2, and in this case E1 was detected as a metabolite. No other metabolites were detected, and EE2 removal started after complete removal of E2, and it increased as the initial E2 concentration increased (Pauwels et al., 2008). *R. erythropolis* and *R. equi* removed EE2 from minimal medium in the presence of co-substrates (adipic acid and glucose). Two metabolites were identified as phenol and a derivative of EE2 with a higher molecular mass (331 atomic mass unit) where the ethinyl group was absent (O’Grady et al., 2009).

In a study by Ma et al. (2018), lab-scale percolation columns were used to investigate EE2 biodegradation during groundwater recharge with reclaimed water. Based on metabolite analysis, different pathways for EE2 degradation were proposed depending on groundwater recharge mode. The dominant pathway in wetting and drying alternative recharge mode started by oxidation of C17 at the D-ring, which is then hydroxylated and cleaved. Thus, EE2 is transformed to E1 → E3 → 7-hydroxy-1-hydroxymethyl-2-methyl-1,2,3,4,4a,9.10,10a-octahydrophenanathrene-2-carboxylic acid → 2-hydroxy-2,4-diene-1, 6-dioic acid. On the contrary, in a continual recharge mode, EE2 metabolism started by hydroxylation of C4 on the A-ring and then the A- or B-ring is cleaved. This transition in the degradation mechanism was proposed to be related to dissolved oxygen and the structure of the involved microbial community.

**Figure 10:**
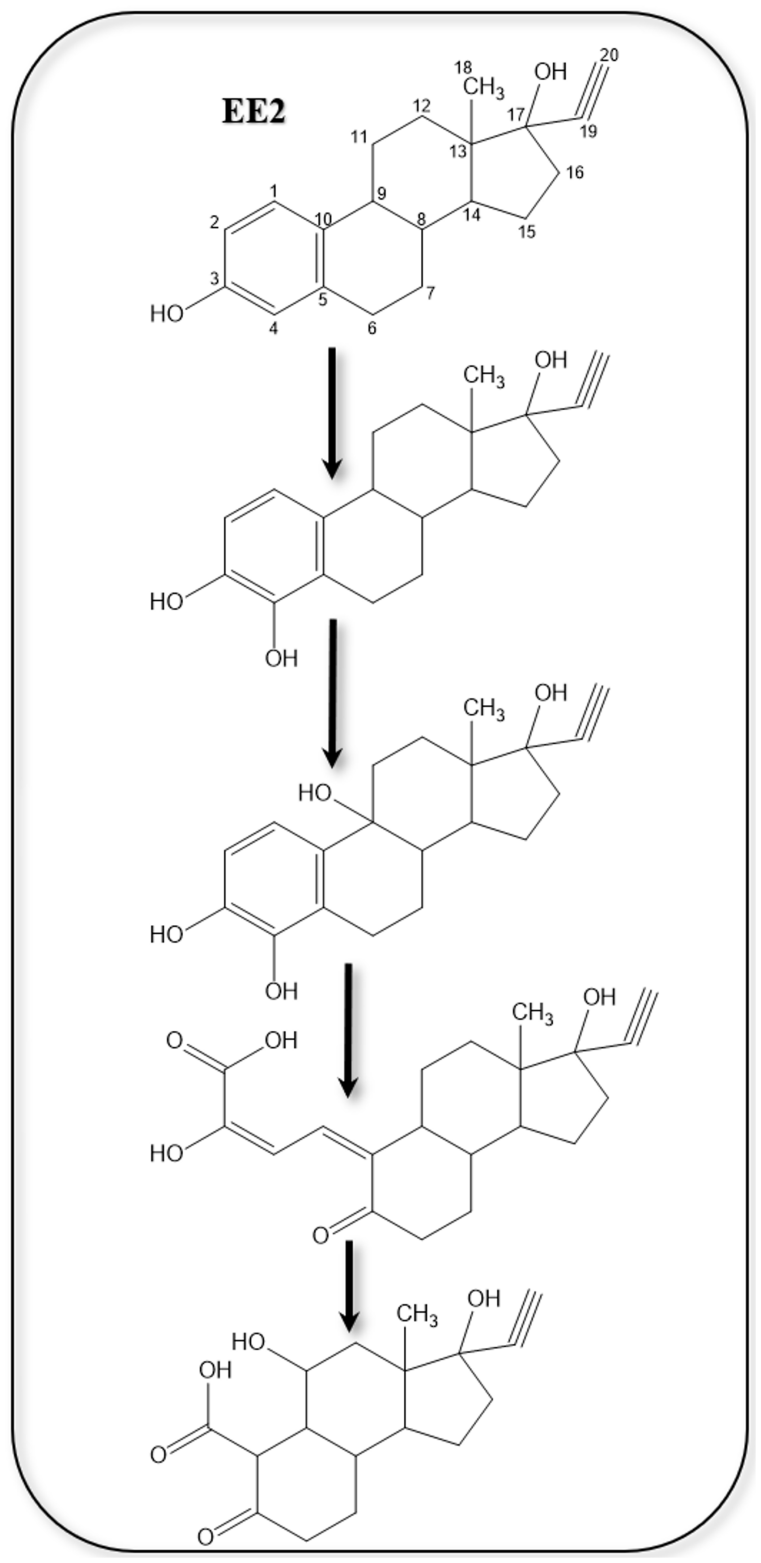
Proposed EE2 metabolites (Wang et al., 2019b).

Recent studies on EE2 degradation in synthetic wastewater by microalgae proposed hydroxylation reactions at C2 or C4, followed by ring opening (Wang et al., 2019b) (Figure 10). However, enzymes and genes of the proposed biochemical reactions were not reported. Peng et al. (2023) studied EE2 degradation by *Pseudomonas citronellolis* SJTE-3 and showed that it can grow on and degrade EE2 only in the presence of co-substrates like ethanol and glucose. The authors also identified two new degradation metabolites. Metabolism of EE2 was inducible, and 8 genes were significantly upregulated in the presence of EE2, including genes encoding a short chain dehydrogenase, a membrane transporter, and a cytochrome P450 hydroxylase, which were crucial for EE2 metabolism.

Guo et al. (2021) studied the effect of rhamnolipid biosurfactants produced by *Pseudomonas aeruginosa* MIG-N146 on the biodegradation of EE2 in shake flasks containing sediment/water systems. The authors reported enhanced biodegradation of EE2 as the rhamnolipid concentration increased, which was attributed to better mass transfer and stimulation of the indigenous microbial consortium. Three metabolic intermediates were detected including a dehydrogenation product of EE2 at C15-C16 of the D-ring, and a carboxylation product of EE2. This study did not report any data on the involved enzymes or microbial community.

Recently, bacteria affiliated with the genera *Aeromonas*, *Rhizobium*, and *Paraburkholderia* isolated from an acid mine drainage degraded EE2 in a minimal medium and produced several degradation metabolites which were mainly ring cleavage products (Palma and Costa, 2024). The authors mentioned that EE2 was the sole carbon source in the cultures. However, EE2 was added from a methanol stock solution, and it was not clear whether it was evaporated before inoculation. Furthermore, while the aim of the study was to isolate EE2-degrading bacterial consortia, all the enrichment and isolation procedures were performed in a rich medium (LB medium) in the presence of paracetamol.

### 4.5 Aerobic E3 degradation

Compared to E1, E2 and EE2, a limited number of studies addressed E3 biodegradation. In most of those studies, E3 was mentioned in the context of substrate spectrum experiments together with other steroid estrogens. Moreover, those studies reported only E3 biotransformation reactions, albeit a complete E3 mineralization pathway is lacking. For instance, Ke et al. (2007) isolated three bacteria from marine sand in microcosms using E1, E2, E3, and EE2 as sole carbon sources. The isolates were identified as members of the genera *Sphingomonas*, *Agromyces*, and *Acinetobacter*. These bacteria, individually or as a consortium, had different substrate spectra in terms of estrogen degradation. The strain identified as *Acinetobacter* sp. degraded E3 in aerobic cultures and transiently produced 16α-OH-E1, which was reported as a metabolite of E2 degradation by sewage bacteria (Lee and Liu, 2002) and of E1 degradation by intestinal bacteria (Järvenpää et al., 1980). Moreover, *Staphylococcus aureus* can reduce 16α-OH-E1 to E3 under anaerobic conditions (Järvenpää et al., 1980). Nitrifying activated sludge and ammonia-oxidizing bacterium *Nitrosomonas europaea* degraded E1, E2, E3 and EE2 in minimal medium (Shi et al., 2004). However, no further information was reported on the degradation mechanisms. Ueda et al. (2012) isolated and characterized a laccase from the mushroom *Pleurotus eryngii* and showed that it can remove E3 from aqueous solutions. A *Comamonas testosteroni* strain isolated from animal waste grew on E3 as a sole carbon and energy source (Liu et al., 2021). Nothing was reported in this study on the specific E3 degradation products, enzymes, or genes. However, genomic analysis revealed the presence of 22 steroid degradation genes. The authors also identified a testosterone degradation gene cluster and a gene encoding 17β-hydroxysteroid dehydrogenase, which catalyzes the transformation of E2 to E1. In the same context, *Rhodococcus* sp. PI4, a marine isolate, utilized E2, E3, and testosterone as sole carbon sources and induced the expression of a 17β-hydroxysteroid dehydrogenase which transforms E3 to 16α-OH-E1 (Ye et al., 2019).

A *Gordonia* sp. strain R9 isolated from chicken leachates utilized E3 and other steroid estrogens as sole carbon sources (Liu et al., 2020). Although no information on E3 degradation was reported, the authors detected E3 as a metabolite of E2. Estriol was also reported as a product of E2 degradation by *Microbacterium resistens* MZT7 (Hao et al., 2022) and *Rhodococcus* sp. RCBS9 (Hao et al., 2024), and it was not clear how E3 is further metabolized.

A study by Hsiao et al. (2021) showed that a *Rhodococcus* sp. strain B50 can grow on E3 as a sole carbon source. However, no further information on the mechanism of E3 degradation was reported. Wang P. et al. (2019) isolated a *Pseudomonas putida* strain SJTE-1 from activated sludge and showed that it can degrade E1, E2, E3 and testosterone as sole carbon sources. Nothing was reported in this study on the E3 degradation mechanisms.

Evidence supporting the conclusion that E3 is degraded by some bacteria via the 4,5-*seco* pathway was reported in recent studies. We showed that a bacterial consortium obtained from raw domestic sewage transformed E3 to E1 potentially via transient formation of 16α-OH-E1. Further degradation of E1 proceeded via the 4,5-*seco* pathway and the community structure of the consortium shifted strongly toward *Croceicoccus estronivorus* which dominated the community after 10 days of incubation (Hashem et al., 2025). Liu et al. (2024) reported the degradation of E3 by *Novosphingobium* sp. ES2-1 and proposed two alternative pathways for E3 degradation based on metabolite analysis. The first proposed pathway proceeds via E3 transformation to E1, which is further degraded via the 4,5-*seco* pathway. However, the authors did not clarify how E3 was transformed to E1. They proposed oxidation and dehydration reactions, albeit no biochemical evidence was presented. The other pathway proposed by Liu et al. (2024) also proceeds by transforming E3 to E1, then to 4-OH-E1, which undergoes B-ring cleavage, resembling the 9,10-*seco* pathway. However, nothing was reported on the genes and enzymes involved in this later pathway. Moreover, the mechanism of the B-ring cleavage was not discussed. The various detected metabolites were not quantified, which is essential to reflect on the relative significance or dominance of the two proposed pathways. Degradation of E3 via the 9,10-*seco* pathway was also proposed by Thathola et al. (2024) using a psychrotolerant *Pseudomonas proteolytica* strain based on the detection of E1, 16-OH-E1, in addition to two proposed ring cleavage downstream products: 2-hydroxy2-4-dienevaleric acid, and 2-hydroxy-2,4-diene-1,6-dioic acid. These products were identified only using mass spectrometry and no further biochemical evidence was presented.

### 4.6 Anaerobic degradation of estrogens

In contrast to the aerobic estrogen degradation, biodegradation of estrogens under anaerobic conditions has been very rarely investigated. The low aqueous solubility (1.5 mg/L at room temperature) of estrogens and stability of the aromatic A-ring render estrogens challenging substrates for microbial utilization (Shareef et al., 2006; Wang et al., 2020). Therefore, under aerobic conditions, bacteria depend on oxygenases and oxygen to activate and cleave the aromatic A-ring through the 4,5-*seco* pathway (Chen et al., 2017; Chiang et al., 2020). Lack of oxygen in anaerobic environments, such as river and marine sediments, prohibits estrogen degradation and makes the process slower compared to aerobic degradation. Hence, anaerobic environments are considered as major reservoirs for estrogens (Hanselman et al., 2003; Czajka and Londry, 2006). Probably, slow degradation rates and difficulties in isolating anaerobic estrogen-degrading bacteria explain why anaerobic estrogen degradation has been less investigated compared to aerobic degradation. Only two bacterial species are known to degrade estrogens under anaerobic conditions, namely, *Denitratisoma oestradiolicum* and *Steroidobacter denitrificans* (Fahrbach et al., 2006, 2008).

To elucidate the biochemical mechanisms of anaerobic estrogen degradation, Wang et al. (2020) isolated a denitrifying bacterium, *Denitratisoma* sp. strain DHT3, from a municipal WWTP and showed that it can efficiently degrade estrogens under denitrifying conditions. The authors sequenced the genome of this strain and performed comparative transcriptomics to identify potential genes involved in anaerobic estrogen degradation using E2- and testosterone-grown cultures. Genes involved in the anaerobic 2,3-*seco* pathway were expressed at similar levels in both cultures. On the contrary, genes of transport, salvage, and reductive activation of cobalamin were upregulated in the E2 culture. In addition, a gene cluster encoding a putative methyltransferase (*emtABCD*) was also upregulated in the E2 culture. Gene disruption experiments confirmed that *emtA* plays a role in anaerobic estrogen degradation by the DHT3 strain.

**Figure 11:**
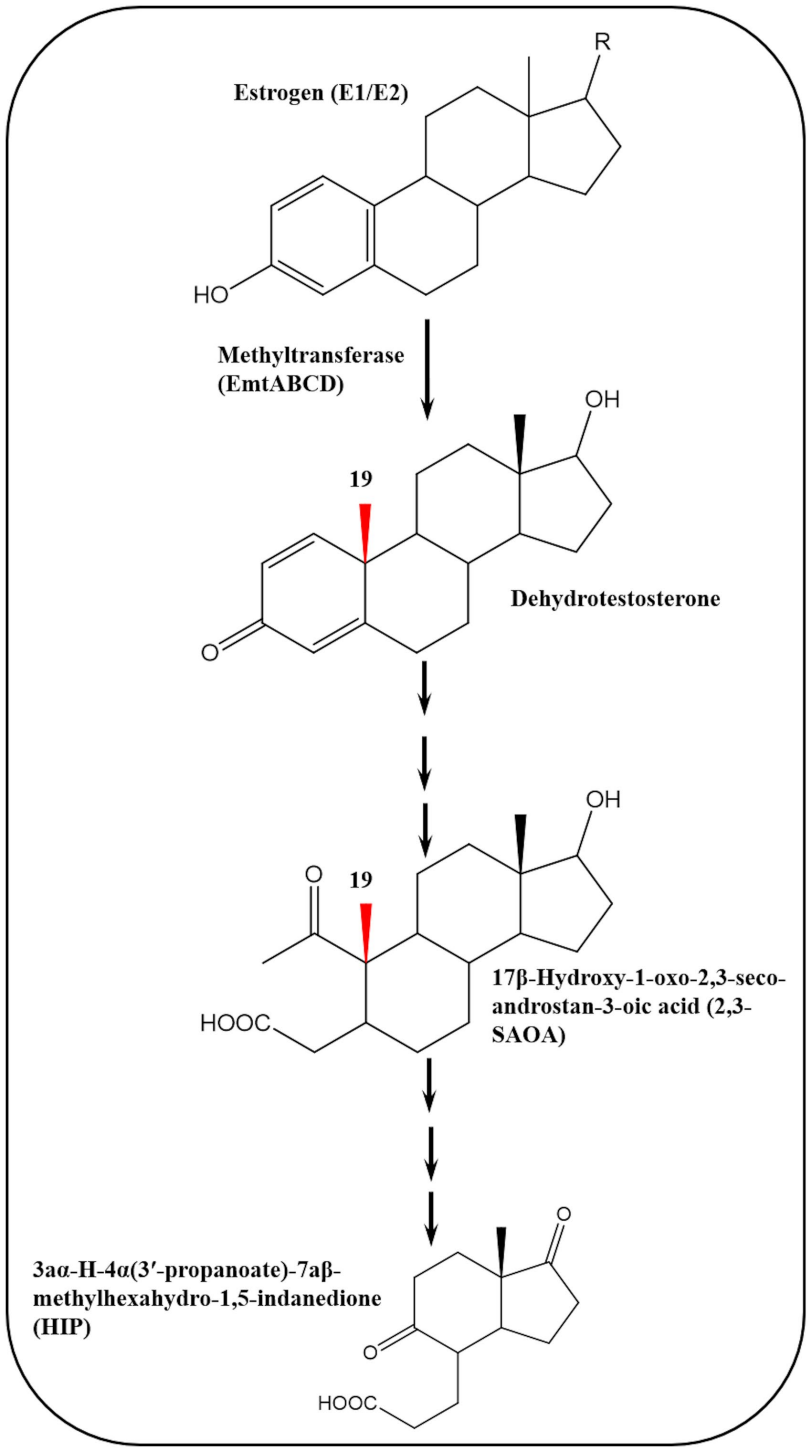
Cobalamin-dependent retroconversion of estrogens into androgens during anaerobic estrogen degradation by *Denitratisoma* sp. strain DHT3 (Wang et al., 2020).

Interestingly, the *emtABCD* gene cluster is present in the genome of estrogen-degrading anaerobes, but lacking in steroid-degrading bacteria that cannot utilize estrogens (Wang et al., 2020). Moreover, metabolite analysis of E1-grown cultures of the DHT3 strain revealed several androgenic metabolites including two ring-cleavage products which are characteristic of the 2,3-*seco* pathway, i.e., 17*β*-hydroxy-1-oxo-2,3-*seco*-androstan-3-oic acid (2,3-SAOA) and HIP. These findings suggested that anaerobic estrogen degradation in the DHT3 strain is initiated by conversion of the C_18_ estrogens to C_19_ androgens which are further degraded via the 2,3-*seco* pathway. The key step is the conversion of estrogens to androgens via a methylation reaction catalyzed by a cobalamin-dependent methyltransferase (*emtABCD*) (Figure 11).

These findings highlight microbe-host metabolic relationships mediated through retroconversion of estrogens into androgens based on the role of sex steroids in bidirectional metabolic interactions between bacteria and their eukaryotic hosts (Karavolos et al., 2013; Markle et al., 2013; vom Steeg and Klein, 2017). Conversion of estrogens into androgens represents the reverse process of estrogen (C_18_) biosynthesis from androgens (C_19_) via removal of the C_19_ angular methyl group, leading to formation of the aromatic A-ring (Miyairi and Fishman, 1985), and it is a thermodynamically challenging reaction which has not been reported in any organism (Wang et al., 2020).

The reverse reaction, namely, the conversion of C_19_ androgens into C_18_ estrogens, was recently discovered in strict anaerobic bacteria (Wang et al., 2025). This aromatase-independent estrogenesis is catalyzed by a cobalamin-dependent methyltransferase (AbeABC), which is 57% identical to the EmtAB methyltransferase identified in the denitrifying *Denitratisoma* spp. Homologs of AbeAB were identified in uncultivated bacteria from marine sediments, suggesting widespread occurrence of anaerobic estrogenesis in anoxic ecosystems. Based on those findings, some ecological implications can be envisaged. For instance, the reversible conversion of estrogens into androgens in anaerobic environments (animal guts and sediments) could potentially affect steroid hormone cycling and fate in these habitats. Furthermore, the unique presence of the *abeAB* genes in microbial genomes render them a functional biomarker that enables monitoring of ecosystem health and tracking steroid pollution (Wang et al., 2025). A recent study showed that EE2 was transformed to E1 and E2 in an electrochemical anaerobic membrane bioreactor (Lei et al., 2025), suggesting that anaerobic degradation of EE2 may proceed via E2 or E1 conversion to androgen. The discovery of this unprecedented steroid microbial bioconversion paves the way for potential diverse applications in environmental monitoring and bioremediation.

The recent studies and findings on anaerobic estrogen biodegradation reflect an increasing interest in this topic whose understanding has been lagging compared to the aerobic degradation processes. Three overlapping drivers can be envisaged to have motivated researchers to investigate this topic further. The first is purely scientific curiosity to understand how bacteria deal with challenging substrates like steroid estrogens in the absence of oxygen or under oxygen fluctuation conditions. Recalling the unprecedented biochemical aspects revealed by extensive studies on the anaerobic degradation of aromatic compounds (Ismail and Gescher, 2012; Boll and Estelmann, 2020; Boll et al., 2020a, 2020b), enables us to reconcile the importance of this driver. The second driver could be the rising global concerns on steroid estrogens as emerging pollutants and the need to better understand their fate and biodegradation mechanisms particularly in anaerobic environments where they tend to accumulate (Hanselman et al., 2003; Czajka and Londry, 2006). While earlier and recent studies addressed the fate and kinetics of estrogen degradation in anaerobic ecosystems (Muller et al., 2010b; Zheng et al., 2012; Luo et al., 2024), unraveling the biochemical mechanisms is pivotal for the development of efficient bioremediation technologies for polluted anaerobic environments due to the recalcitrance of estrogens in the (Pratush et al., 2020a). The third driver is related to the overwhelming recent interest in understanding the functional relevance of the gut microbiome in health and pathogenesis, which is instrumental toward the development of novel therapies and probiotics (Li et al., 2022; Diviccaro et al., 2025). In this context, it is known that sex steroids mediate bidirectional interactions between bacteria and their eukaryotic hosts (Kverka and Stepan, 2024; Nieto et al., 2025). Moreover, bacteria can alter a host’s sex steroid profile through interconversions (biotransformations), utilization as a carbon and electron source, and affecting endogenous estrogen metabolism in postmenopausal women (Horinouchi et al., 2012; Markle et al., 2013; Ridlon et al., 2013; Fuhrman et al., 2014; Zhang et al., 2025). While the three proposed drivers guide research in different directions with different objectives, they share better understanding of the subject matter as a key enabler and will continue to fuel research on estrogen microbial metabolism and associated applications in health care and environmental biotechnology.

### 4.7 Central pathway of bacterial estrogen degradation

**Figure 12:**
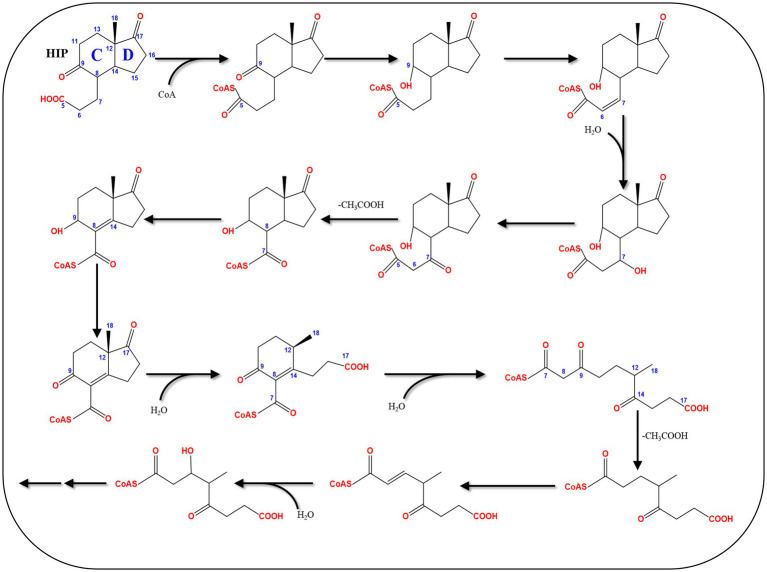
The HIP degradation pathway (Chiang et al., 2020).

A C_13_ metabolite (HIP), containing the C/D rings of the steroid substrate, was commonly identified in all bacterial pathways for steroid catabolism. Hence, it is degraded through a common central catabolic pathway which targets the C/D rings (Table 3; Figure 12). After activation by a specific acyl-CoA synthetase (FadD3), the remaining carbons of the steroid B-ring are removed through β-oxidation. This is followed by hydrolytic cleavage the D-ring by an enoyl-CoA hydratase (EchA20), and another hydrolase (IpdAB) opens the C-ring. Interestingly, gene mining studies revealed the presence of conserved gene clusters for HIP degradation in the majority of experimentally verified steroid-degrading bacteria (Chiang et al., 2020).

### 4.8 Abiotic degradation and transformation of estrogens

In addition to biological degradation and transformation, estrogens are exposed to several abiotic factors in the environment, which can lead to some degradation and transformation reactions, including chemical and photodegradation/transformation. These abiotic reactions may affect the fate of estrogens in the environment and can also directly interfere with the susceptibility of estrogens to biodegradation and biotransformation (Griffith et al., 2023). This depends on the nature of the products of the abiotic reactions, which could be more prone to biodegradation or more recalcitrant than the estrogen substrate. For instance, chlorine is commonly applied as a disinfectant during water treatment due to its strong oxidation capability, continuous disinfection, and low cost (Deborde and von Gunten, 2008). However, chlorine often produces undesirable intermediate byproducts and potentially carcinogenic halogenated byproducts (Richardson and Kimura, 2016).

Estrogen degradation during chlorination was reported mostly in lab-scale studies, showing the formation of intermediate products and disinfection byproducts (Deborde et al., 2004; Lee and von Gunten, 2009). At an initial concentration of 50 mg/L, E2 was completely degraded after 10 min during chlorination at pH 7.5 (Hu et al., 2003). In batch tests, E3 degradation during chlorination followed a second-order kinetic (Zhao et al., 2014). Moreover, E1 degradation with free chlorine produced chlorinated estrone by-products (Nakamura et al., 2007). The kinetics and products of E2 chlorination were also studied in a pilot-scale water distribution system (He et al., 2016; Li et al., 2017). Further studies by Li et al. (2017) on the kinetics and mechanism of E2 chlorination in a pilot-scale water distribution system showed that chlorination degraded E2 in three stages: (1) halogenation of the aromatic ring, (2) cleavage of the benzene moiety and chlorine substitution, (3) production of trihalomethanes and halogenated acetic acids from the phenolic intermediates.

**Figure 13:**
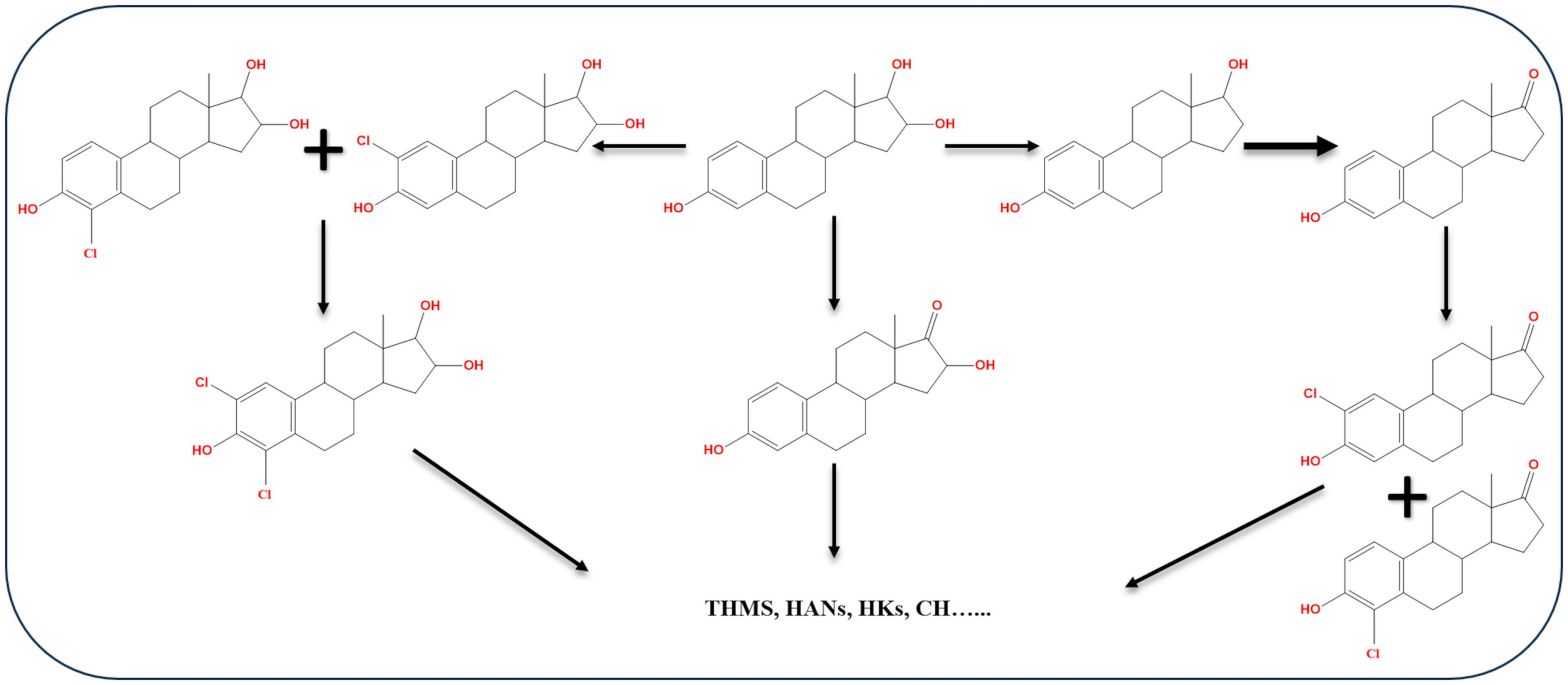
Proposed E3 chlorination pathways. THMs, trihalomethane; HANs, haloacetonitrile; HKs, haloketone; CH, chloral hydrate (Dong et al., 2019).

During E3 chlorination in a pilot-scale water distribution system, E2, E1, 16α-OH-E1, and chlorinated E3, in addition to four types of disinfection by-products including trihalomethanes, haloacetonitriles, haloketones, and chloral hydrate were identified as intermediate products (Dong et al., 2019). Based on the identified products, three possible pathways for E3 chlorination were proposed (Figure 13). During water chlorination, EE2 was transformed into 4-chloro-EE2 and 2,4-dichloro-EE2. In the presence of Br^−^, the bromo-analogs of the chloro-EE2 derivatives were additionally produced (Lee and von Gunten, 2009). In drinking water, chlorination of E2 produced several transformation products including 2,4-dichloro-E2, monochloro-E1, 2,4-dichloro-E1, and by-product such as 4-[2-(2,6-dichloro-3-hydroxyphenyl)ethyl]-7α-methyloctahydroinden-5-one (Hu et al., 2003).

Chlorinated E1, such as 2-chloro-E1, 4-chloro-E1, 2,4-dichloro-E1, were detected in a sewage treatment plant effluent in Japan. Moreover, in a buffer of pH 7 and 9 combining E1 with sodium hypochlorite, the same chlorinated products were produced in addition to 1,4-estradiene-3,17-dione derivatives resulting from the reaction of 2,4-dichloro-E1 and sodium hypochlorite (Nakamura et al., 2007). Ten oxidation products of E2 were identified during treatment with chloramine in a water distribution system. The identified products were formed by addition or loss of a functional group at different positions. The proposed reactions include electrophilic substitution of the ortho-position of the phenol moiety, elimination reaction between C9 and C11, and a hydrogenation reaction on the benzene ring (He et al., 2016). In general, biodegradation studies on halogenated estrogens are very scarce. Recently, the findings of Griffith et al. (2023) on biodegradation kinetics of free and halogenated estrogens in river water-sediment microcosms suggested that halogenated estrogens degrade more slowly than the corresponding free estrogens. Halogenated E1 derivatives were detected as transformation products of halogenated E2. While detection of halogenated E1 derivatives suggests subsequent metabolism via the most common 4,5-*seco* pathway, further studies are needed to unravel how dehalogenation of the A-ring occurs before hydroxylation and ring cleavage. Moreover, biodegradation kinetics and pathways in wastewater microcosms containing a mixture of free and halogenated estrogens is worth investigating.

Beside chlorination, advanced oxidation processes are being investigated as a wastewater treatment to eliminate endocrine-disrupting chemicals including estrogens. These processes include ozonation, photocatalysis, Fenton and Fenton-like processes, and they depend on the production of highly reactive hydroxyl radicals to oxidize organic pollutants (Wang and Wang, 2016; Dewil et al., 2017). There is also an interest in the development of chemical-free electrochemical oxidation processes (Dewil et al., 2017). As these processes depend on very strong oxidizing agents, namely hydroxyl radicals, they can lead to several hydroxylated and ring-cleavage transformation products. Even the ethinyl group of EE2 can be removed (Reis et al., 2023).

In the environment, estrogens are prone to direct and indirect photo-transformation reactions, which can play a role in estrogen removal (Chen et al., 2013; Adeel et al., 2017). The most common photo-transformation products of estrogen are hydroxylated derivatives bearing hydroxy groups at various positions (Mazellier et al., 2008; Li Puma et al., 2010; Caupos et al., 2011; Chen et al., 2013; Zhao et al., 2019). In addition, some ring cleavage products were also reported during estrogen photodegradation (Perondi et al., 2020). Susceptibility of estrogens to photodegradation can be exploited to develop concerted photocatalysis-biocatalysis technologies that can enhance estrogens removal, as shown for conjugated E2. Photocatalysis can contribute to deconjugation transformations which make free estrogens available for microbial degradation (Ding et al., 2024).

## 5 Concluding remarks and future research directions

Steroid estrogens are endocrine-disrupting chemicals that can be harmful to public health and the aquatic environment at extremely low concentrations. It has been recognized that effluents from WWTPs are major source of environmental pollution with estrogens. Hence, proper treatments of wastewater are instrumental to avoid environmental pollution with these hazardous compounds. Microbial biodegradation has been studied as a key process for the elimination of estrogens from wastewater. Despite the very low aqueous solubility and relatively inert chemical structure, several bacteria are endowed with metabolic capabilities to utilize these challenging substrates as a carbon and energy source for growth, while others can only perform specific biotransformations on estrogens. Estrogen-degrading bacteria were isolated from engineered and natural environments such as activated sludge, soil, compost, and sandy aquifers, and the majority of them belong to Actinomycetota and Pseudomonadota. Most studies focused on the aerobic degradation of E1 and E2, and the involved enzymes, genes and pathways were investigated in some detail. On the contrary, EE2 was much less studied and even fewer studies are available for E3. This may be due to the very high structural similarity among the different estrogens, which could lead to the perception that E3 and EE2 biodegradation may proceed similarly to E1and E2. Furthermore, the relatively high recalcitrance of EE2, due to the ethinyl group, rendered the isolation of EE2-degrading microbes a challenge. However, the large diversity of the reported biotransformation reactions and biodegradation pathways justifies more in-depth studies to unravel specific biodegradation mechanisms and key degrading microbes for E3 and EE2.

Despite the high structural similarity between steroid estrogens, diverse estrogen degradation pathways have been reported. A common feature of those pathways is the start by modification of a certain ring of the estrogen substrate followed by cleavage of that ring. However, for most of the proposed degradation pathways or biotransformation reactions, biochemical and molecular evidence at the level of the involved enzymes and genes is lacking. Exceptionally, only the 4,5-*seco* pathway of aerobic estrogen degradation has been thoroughly investigated. Interestingly, biochemical and molecular studies on the 4,5-*seco* pathway revealed some differences between Actinomycetota and Pseudomonadota, which can be exploited as functional biomarkers for environmental and ecological assessment of estrogen biodegradation. Anaerobic estrogen degradation has been rarely studied, and key mechanisms have been only recently elucidated, indicating that anaerobic estrogen degradation proceeds through the 2,3-*seco* pathway after a unique initial retro conversion of estrogens into androgens.

**Figure 14:**
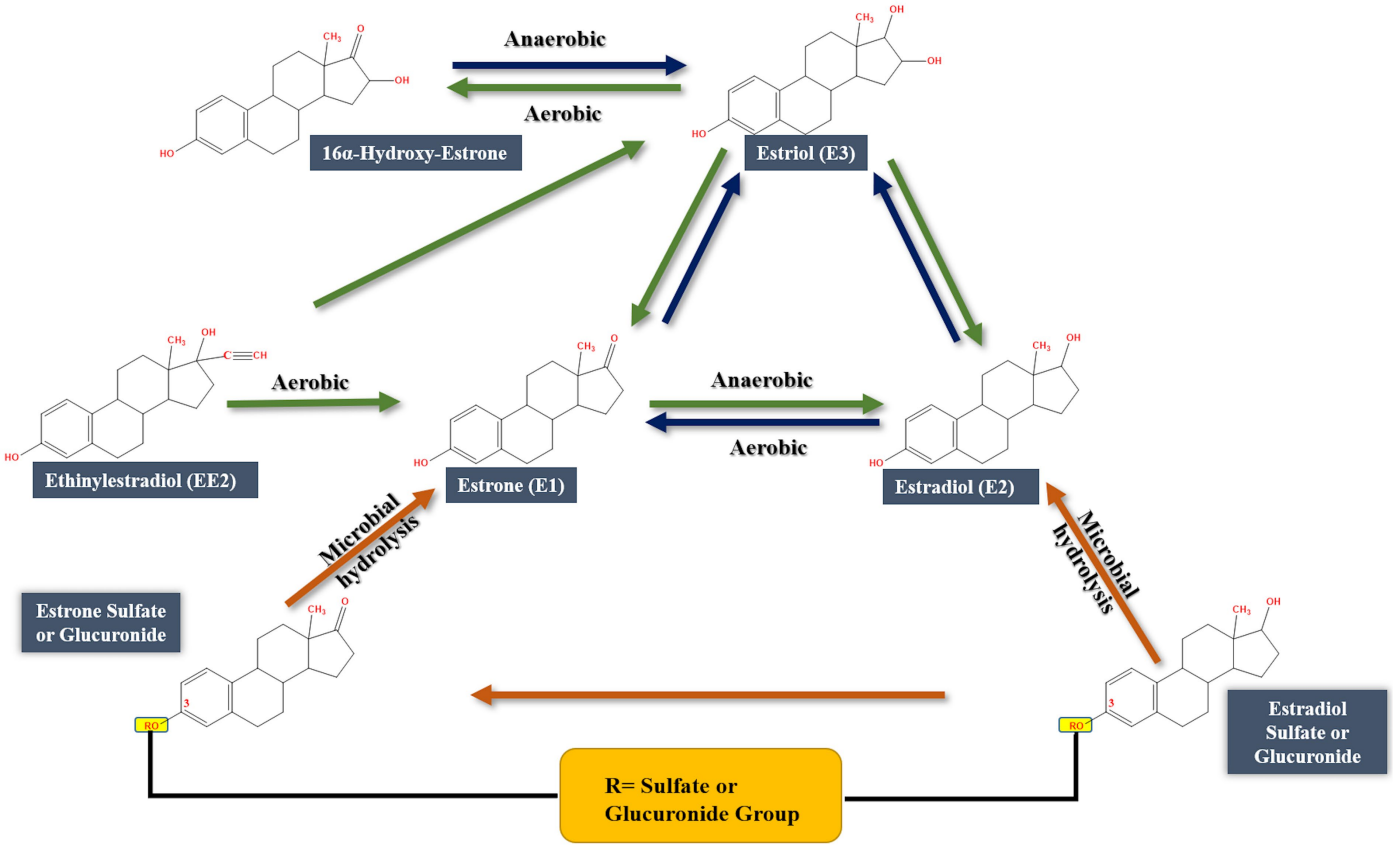
Interconversions between different steroid estrogens (Adeel et al., 2017; Yu et al., 2019).

Regardless of oxygen availability, both the aerobic and anaerobic pathways converge at a common central metabolite of steroid catabolism by bacteria; namely, HIP. The latter is then degraded through a common central metabolic pathway. One of the characteristic features of estrogen biodegradation is the interconversion of one compound to another as shown in Figure 14. This feature might have some implications on the environmental fate of estrogens due to changes in their concentrations and potential substrate preference differences among the involved microbial communities, particularly in the presence of estrogen mixtures. In addition, abiotic degradation of estrogens occurs in the environment via photochemical reactions which can potentially impact their susceptibility to biodegradation/biotransformation process. Nonetheless, biodegradation of the products of abiotic estrogen degradation and transformation have been scarcely investigated.

Studies on estrogen biodegradation applied mostly pure cultures and single estrogen substrates, which is not necessarily environmentally relevant where there is a mixture of estrogens and other carbon sources, and microbes exist as complex and heterogeneous communities. Moreover, there are still many knowledge gaps on the mechanisms and microbes involved in E3 and EE2 biodegradation. It was clear that E3 and EE2 need further studies to unravel their degradation mechanisms and the involved microbial communities. Furthermore, future research should focus more on microbial consortia as they are prevailing in natural and engineered ecosystems. However, it is crucial to investigate the functionality of isolated microbial consortia in polluted environmental matrices such as raw sewage and activated sludge. Moreover, stability of estrogen-degrading microbial consortia in terms of functional resilience/resistance needs to be properly addressed. In addition, more work is needed to elucidate estrogen biodegradation process in environmental matrices to gain more realistic and conclusive data for *in-situ* applications. A holistic or systems approach, encompassing proteomics, transcriptomics, metabolomics, comparative genomics, metagenomics, and metaproteomics, can be instrumental in future studies. It is also crucial to unravel how estrogen uptake occurs in estrogen-degrading microbes as knowledge of estrogen uptake systems is largely lacking. The impact of abiotic estrogen degradation on the biological treatment of estrogen-polluted environments deserves proper attention as it might augment or prohibit the biodegradation/biotransformation processes. Some limitations of most of the available studies need to be rectified in future. The use of more environmentally relevant estrogen concentrations, the use of estrogen mixtures (with and without other steroids), and the effect of non-steroid co-substrates are of particular interest.

Despite active research on estrogens–degrading bacteria and accumulation of knowledge during recent years, it is obvious that we have just scratched the surface of this field. Further intensive research is needed on different aspects of estrogens biodegradation. This is a pre-requisite to comprehensively understand the underlying mechanisms and elucidate the structural and functional diversity of estrogens-degrading microbial communities, which should enable efficient estrogens removal from engineered and natural polluted environments via implementation of well-designed bioremediation technologies.
