# Supplementary material for: Diversity of estrogen biodegradation pathways and application in environmental bioremediation
Source: Front Microbiol. 2025 Sep 25;16:1630636. doi: 10.3389/fmicb.2025.1630636 (PMC12507874; doi:10.3389/fmicb.2025.1630636)
Supplement: Supplementary file 1 [file Data_Sheet_1.docx]

**Supplementary Material**

**Diversity of Estrogen Biodegradation Pathways and Application in Environmental Bioremediation**

Jaleela S. Hashem^1^, Wael Ismail^2*^, Yin-Ru Chiang^3^, Adnan A. Bekhit^1,4^

^1^Allied Health Sciences Department, College of Health and Sport Sciences, University of Bahrain, P.O. Box 32038, Bahrain

^2^Center of Environmental and Biological Studies, Arabian Gulf University, Bahrain

^3^Biodiversity Research Center, Academia Sinica, Taipei, Taiwan

^4^Department of Pharmaceutical Chemistry, Faculty of Pharmacy, Alexandria University, Alexandria 21521, Egypt

^*^Corresponding author: Tel: +973-36146948; E-mail: [waelame@agu.edu.bh](mailto:waelame@agu.edu.bh)

**Table S1.** List of some estrogen-degrading bacteria isolated from activated sludge (Modified from Flint, 2019)

| Bacterial species | Estrogen substrates |
| --- | --- |
| *Novosphingobium tardaugens* sp. ARI-1 | Metabolizes E1, E2 and E3 |
| *Aminobacter aminovorans* sp. KC7 | Degrades* E1 and E2 |
| *Aminobacter* sp. KC6 | Degrades* E1 and E2 |
| *Sphingomonas* strain KC8 | Metabolizes E1 and E2 |
| *Brevundimonas diminuta* strain NK 2 (EU35276) | Transforms E2 to E1 |
| *Brevundimonas vesicularis* KC12 | Transforms E2 to E1 |
| *Rhodococcus ruber* M2 KC4 | Transforms E2 to E1 |
| *Microbacterium testaceum* KC5. | Transforms E2 to E1 |
| *Sphingomonas* sp. KC9-11 | Transforms E2 to E1 |
| *Flavobacterium* sp. KC1 | Transforms E2 to E1 |
| Bacteroidetes sp. KC2 | Transforms E2 to E1 |
| *Nocardioides simplex* sp. KC3 | Transforms E2 to E1 |
| *Escherichia coli* sp. KC13 | Transforms E2 to E1 |
| *Sphingomonas* sp. KC14 | Transforms E2 to E1 |
|  |  |
| *Achromobacter xylosoxidans* | Degrades* E1, E2 and  transforms E3. |
| *Ralstonia* sp. | Degrades* E1, E2 and  transforms E3. |
| *Pseudomonas aeruginosa* sp. TJ1 | Metabolizes E2. |
| *Bacillus subtilis* sp. E2Y4 | Degrades* E1 and E2 |
| *Bacillus* sp. E2Y1 | Degrades* E1 and E2 |
| *Bacillus amylonquefaciens* sp. E2Y2 | Transforms E2 to E1 |
| *Bacillus* sp. E2Y3 | Transforms E2 to E1 |
| *Bacillus cereus* sp. E2Y5 | Transforms E2 to E1 |
| *Denitratisoma oestradiolicum* gen. nov., sp. nov | Metabolizes E2 under anoxic conditions |
| *Steroidobacter denitrificans* gen. nov., sp. nov | Metabolizes E2 under anoxic conditions |
| *Nitrosomonas europaea* | Co-metabolizes E1, E2, E3 and EE2 under nitrifying conditions |
| *Sphingobacterium* sp. JCR5 | Metabolizes E1, E2, E3 and EE2. |
| *Novosphingobium* sp. JEM-1 | Degrades* E1 and E2 |
| *Rhodococcus zopfii* strain Y 50158 | Metabolizes E1, E2, E3 and EE2 |
| *Rhodococcus equi* strains | Metabolizes E1, E2, E3 and EE2 |
| *Pseudomonas citronellolis* SS-2 | Degradation of E1, E2 and  EE2, but not E3 |
| *Pseudomonas citronellolis* SJTE-3 | Metabolizes E1, E2, E3 and EE2 |
| *Novosphingobium* sp. E2S | Degradation of E2 |
| *Rhodococcus* sp. DS201 | Metabolizes E2 and E1 |

* Where strains were unable to grow upon estrogen as the sole energy or carbon source, but co-metabolism was not confirmed. All strains are aerobic unless otherwise stated.

**Table S2.** List of known estrogen-degrading bacteria isolated from various sources (Modified from Flint, 2019)

| Bacteria | Substrates | Source of bacteria |
| --- | --- | --- |
| *Phyllobacterium myrsinacearum* | Metabolizes E1, E2, E3 and co-metabolizes EE2 in the presence of E1, E2 and E3 | Compost |
| *Ralstonia picketti* BP2 | Metabolizes E1, E2, E3 and co-metabolizes EE2 in the presence of E1, E2 and E3 | Compost |
| *Pseudomonas aeruginosa* BP3 | Metabolizes E1, E2, E3 and co-metabolizes EE2 in the presence of E1, E2 and E3 | Compost |
| *Pseudomonas* sp. BP7 | Metabolizes E1, E2, E3 and co-metabolizes EE2 in the presence of E1, E2 and E3 | Compost |
| *Acinetobacter* sp. | Metabolizes E1, E2, E3 and co-metabolizes EE2 in the presence of E1, E2 and E3 | Compost |
| *Sphingomonas* sp. CYH | Degrades E1 (aerobic and anoxic) and transforms E2 to E1 | Artificial sandy  aquifer |
| *Agromyces* sp. LHJ3 | Degrades E3 (aerobic), E2 (anoxic) and transforms E2 to E1 | Artificial sandy  aquifer |
| *Acinetobacter* sp. LHJ1 | Transforms E2 to E1 | Artificial sandy  aquifer |
| *Sphingomonas* ED8 and 9. | Metabolizes E1 and E2 | Soil |
| *Rhodococcus* ED6, 7 and 10. | Metabolizes E1 and E2 | Soil |
| *Pseudomonas putida* SJTE-1 | Metabolizes E1 and E2 | Soil |
| *Leptothrix discophora* | Manganese Mn2+- dependent degradation of EE2 | Belgian coordinated  collections of microorganisms |
| *Pseudomonas putida* MnB1, 6 and 29. | Manganese Mn2+- dependent degradation of EE2 | Belgian coordinated  collections of microorganisms |
| *Nitrosomonas europaea* ATCC 19718 | Co-metabolism and Nitration of EE2 | ATCC |
| *Buttiauxella* | Metabolism of E2 and Testosterone | Baltic Sea |
| *Vibrio* sp. H5 | Metabolism of E2 and Testosterone | Baltic Sea |
| *Rhodococcus equi* ATCC 13557 | Partial degradation of EE2 in the presence of a cosubstrate EE2 61% ± 1% in 300h | ATCC |
| *Rhodococcus erythropolis* ATCC 4277 | Partial degradation of EE2 in the presence of a cosubstrate EE2 46% ± 2% in 300 h | ATCC |
| *Rhodococcus zopfii* ATCC 51349 | Partial degradation of EE2 in the presence of a cosubstrate EE2 38% ± 1% in 300h | ATCC |
| *Bacillus subtilis* ATCC 6051 | Partial degradation of EE2 in the presence of a cosubstrate EE2 27% ± 2% in 300h | ATCC |
| *Pseudomonas aeruginosa* PA01 | Partial degradation of EE2 in the presence of a cosubstrate EE2 34% ± 2% in 300h | ATCC |
| *Pseudomonas putida* ATCC12633 | Partial degradation of EE2 in the presence of a cosubstrate EE2 21% ± 2% in 300h | ATCC |
| *Rhodococcus rhodochrous* ATCC13808 | Partial degradation of EE2 in the presence of a cosubstrate No EE2 detected after 48 h  (possibly co-metabolic) | ATCC |
| *Bacillus cereus* Socransky 67 | Conversion of E2 to unknown metabolites | ATCC |
| *Streptococcus mutans* | Conversion of E2 to E1 | Dental plaque |
| *Streptococcus mutans* NCTC 10449 | Conversion of E2 to E1 | Dental plaque |
| *Streptococcus mutans* NCTC 10904 | Conversion of E2 to E1 | Dental plaque |
| *Rhodococcus sp. P14* | Conversion of E2 to E1 | Dental plaque |
| *Nocardia sp.* E110 | Degradation of E1 | Crude oil |

ATCC: American Type Culture Collection

**References**

Flint, S. L. (2019). The genomic evaluation of estrogen degradation by *Rhodococcus equi* ATCC 13557. <http://theses.ncl.ac.uk/jspui/handle/10443/4513>
